# Stafiba: A STAT5‐Selective Small‐Molecule Inhibitor

**DOI:** 10.1002/cbic.202200553

**Published:** 2022-11-24

**Authors:** Katrin S. Eckhardt, Theresa Münzel, Julian Gräb, Thorsten Berg

**Affiliations:** ^1^ Leipzig University Institute of Organic Chemistry Johannisallee 29 04103 Leipzig Germany

**Keywords:** biological activity, inhibitors, protein-protein interactions, SH2 domains, transcription factors

## Abstract

The transcription factors STAT5a and STAT5b are constitutively active in many human tumors. Combined inhibition of both STAT5 proteins is a valuable approach with promising applications in tumor biology. We recently reported resorcinol bisphosphate as a moderately active inhibitor of the protein‐protein interaction domains, the SH2 domains, of both STAT5a and STAT5b. Here, we describe the development of resorcinol bisphosphate to Stafiba, a phosphatase‐stable inhibitor of STAT5a and STAT5b with activity in the low micromolar concentration range. Our data provide insights into the structure‐activity relationships of resorcinol bisphosphates and the corresponding bisphosphonates for use as inhibitors of both STAT5a and STAT5b.

## Graphical abstract

**Dual inhibition** of the transcription factors STAT5a and STAT5b is a valuable approach with promising applications in tumor biology. We describe the development of resorcinol bisphosphate to Stafiba, a phosphatase‐stable inhibitor of STAT5a and STAT5b with activity in the low micromolar concentration range.

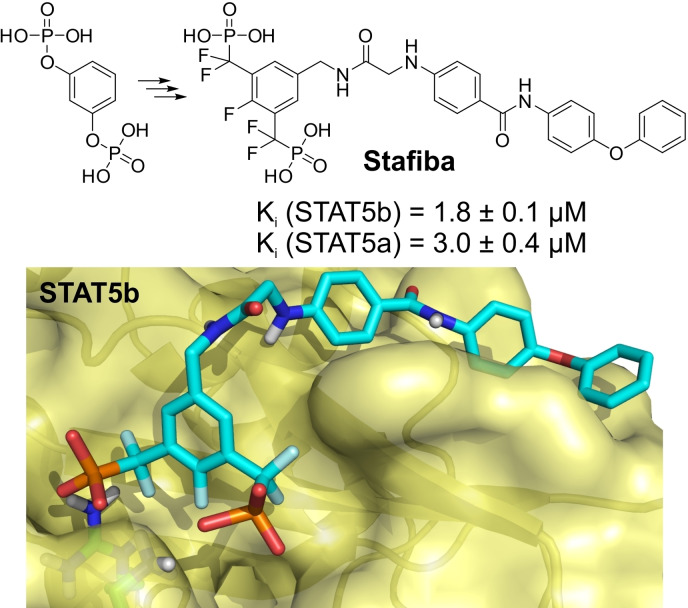

## Introduction

**Figure 1:**
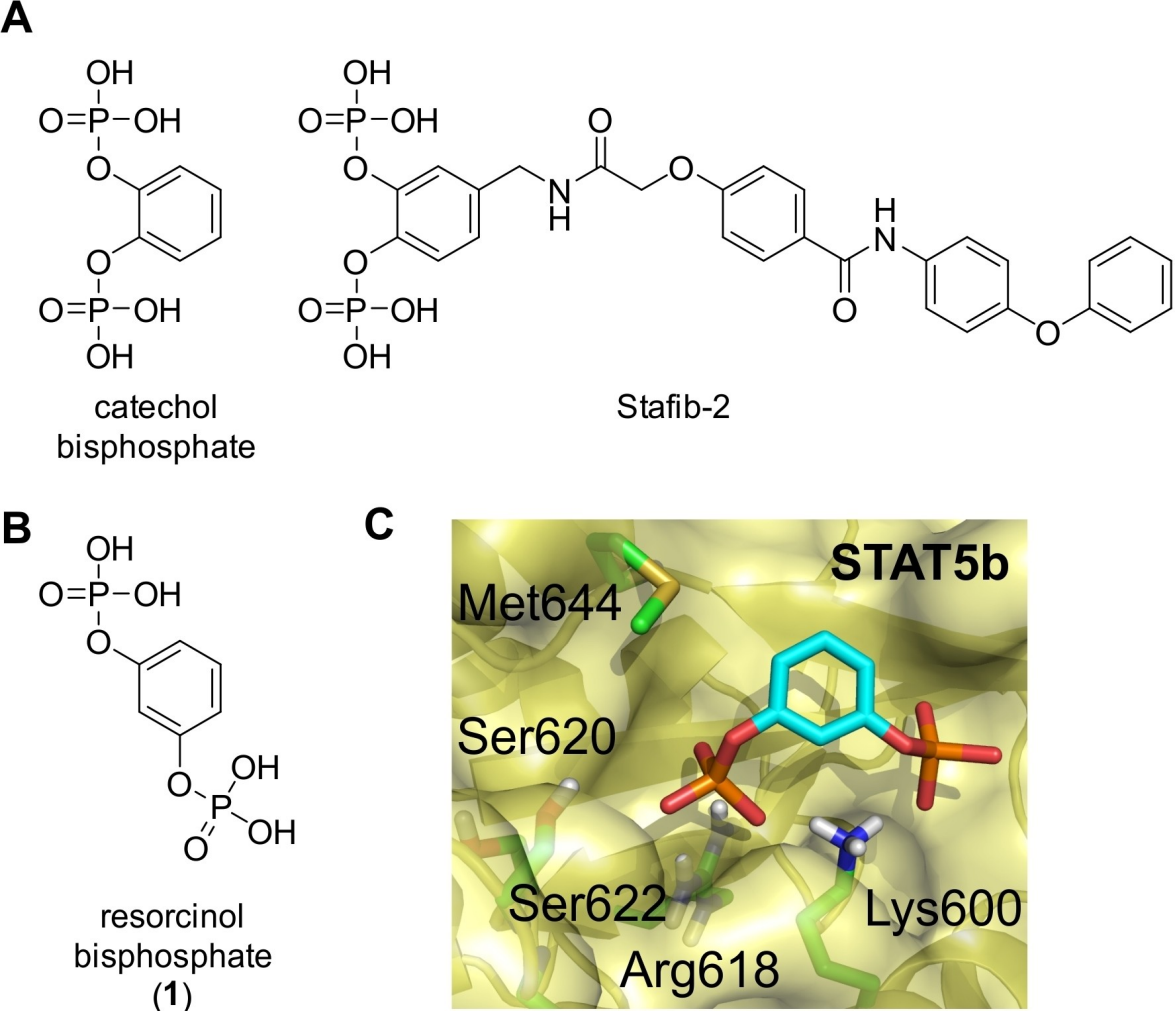
Structures of A) catechol bisphosphate and Stafib‐2, and B) resorcinol bisphosphate (**1**). C) Binding mode of **1** to STAT5b as suggested by AutoDockFR. The Figure was created using PyMOL.^[8]^

The transcription factors STAT5a and STAT5b are being constitutively activated in multiple human tumor types.^[1]^ Despite their high degree of sequence homology (96 % on the protein level), they have both redundant and non‐redundant functions.^[2]^ While STAT5b has been identified as the main driver of cell growth and tumorigenesis,[^2e^, ^2f^, ^2g^, ^2h^, ^2i^, ^2j^] the ability of STAT5a to compensate for STAT5b in many functions makes a supporting role for STAT5a during tumorigenesis likely. Therefore, combined inhibition of both STAT5 proteins is a valuable approach with promising applications in tumor biology. For the majority of inhibitors shown to directly inhibit STAT5 in an *in vitro* assay, specificity for one STAT5 protein over the other has not been reported.^[3]^ We recently presented *m*‐terphenyl phosphates as the first selective inhibitors of STAT5a,^[4]^ and catechol bisphosphate and optimized derivatives such as Stafib‐2 as the first selective inhibitors of STAT5b (Figure 1A).^[5]^ To a lesser degree, selectivity for STAT5b over STAT5a was also observed for fosfosal^[6]^ and purine nucleotide triphosphates.^[7]^

## Results and Discussion

In the course of discovery of catechol bisphosphates as selective STAT5b inhibitors, we had also reported resorcinol bisphosphate (**1**) as a moderately active inhibitor of both STAT5 proteins in fluorescence polarization (FP) assays [K_i_ (STAT5a)=23.3±4.9 μM, K_i_ (STAT5b)=20.4±0.7 μM, Figure 1B, Tables 1 and S1].^[5a]^ In this study, we aimed to improve the activity of resorcinol bisphosphate and to convert the most potent resorcinol bisphosphate derivative to a phosphatase‐stable bisphosphonate.

**Table 1:** Structures and activities of test compounds against STAT5a and STAT5b in FP assays. K_i_ values were calculated from IC_50_ values (n=3) via the published equation.^[13]^

No	Structure	K_i_ [μM] (STAT5a)	K_i_ [μM] (STAT5b)
**1**	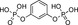	23.3±4.9	20.4±0.7
**2**	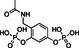	n/a	42.1±1.6
**3**	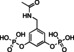	36.4±3.8	18.7±0.8
**4**	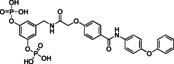	2.5±0.5	0.9±0.1
**5**	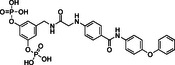	3.1±0.1	1.5±0.1
**6**	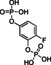	29.4±0.6^[a]^	27.4±2.8
**7**	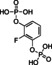	10.8±0.5	7.6±0.4
**8**	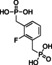	n/a	n/a
**9**	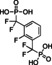	15.0±0.6	18.9±1.5
**10**	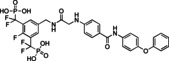	3.0±0.4	1.8±0.1

[a] n=2. n/a: not applicable.

Using AutoDockFR,^[9]^ we performed molecular docking of resorcinol bisphosphate into our previously published homology model of STAT5b,^[6a]^ based on the X‐ray structure of STAT5a.^[10]^ This suggested interactions of the phosphoryl groups of **1** with both Lys600 and Arg618 in the Src homology 2 (SH2) domain (Figure 1C), the protein‐protein interaction domain, of STAT5b. The corresponding amino acid positions are conserved in all STAT proteins and interact with the phosphate group of phosphotyrosine‐bearing binding peptide sequences.^[11]^ Thus, the putative binding pocket of resorcinol bisphosphate (**1**) is very close to the binding pocket of catechol bisphosphate in STAT5b, which is located between Lys600 / Arg618 in the SH2 domain, and Arg566 in the adjacent linker domain.^[12]^ STAT5a carries a tryptophan in position 566, which provides an explanation for the selectivity of catechol bisphosphates for STAT5b over STAT5a.^[12]^ The absence of specificity of **1** for one STAT5 protein over the other suggests that the divergent linker domain amino acids in position 566 of STAT5a/STAT5b do not play a significant role for binding of **1**.

In the process of optimization of catechol bisphosphate to the more active derivatives Stafib‐1^[5a]^ and Stafib‐2,^[5c]^ we had noticed that the STAT5b‐selectivity originated from the hydrophilic catechol bisphosphate core, because its extension with relatively hydrophobic moieties increased the compounds’ selectivity for STAT5b over STAT5a only moderately. In consequence, we hypothesized that resorcinol bisphosphate could be extended with the same hydrophobic building block as contained in the most potent STAT5b inhibitor Stafib‐2 to obtain high affinity, dual inhibitors of STAT5a/5b. To find a suitable attachment point for the hydrophobic part of Stafib‐2, we first extended resorcinol bisphosphate with an acetamidomethyl group, either in the 4‐position (compound **2**, Tables 1 and S1) or in the 5‐position (compound **3**). Placement of the acetamidomethyl group in the 4‐position (compound **2**) turned out to be less suited [50±3 % inhibition of STAT5a at 100 μM, the highest concentration tested, and K_i_ (STAT5b)=42.1±1.6 μM, Tables 1 and S1)], than placement in the 5‐position (compound **3**), which left the activities [K_i_ (STAT5a)=36.4±3.8 μM, K_i_ (STAT5b)=18.7±0.8 μM, Tables 1 and S1)] relatively unchanged as compared to those of resorcinol bisphosphate (**1**). In consequence, we synthesized compound **4**, in which the more hydrophobic part of Stafib‐2 is connected to the 5‐position of resorcinol bisphosphate (**1**) (Table 1, Figure S1). This step increased the activity against STAT5a by 9‐fold (K_i_=2.5±0.5 μM), and against STAT5b by 23‐fold (K_i_=0.9±0.1 μM, Tables 1 and S1), shifting the selectivity profile towards STAT5b by approximately twofold. However, synthesis of **4** was hampered by low yields, which could be attributed in part to an unwanted cleavage of the central arylether function in the course of the synthesis (Figure S1), and the limited solubility of the final product. We hypothesized that both aspects could be improved by replacing the central arylether by a secondary amine, as represented by compound **5**.

**Figure 2:**
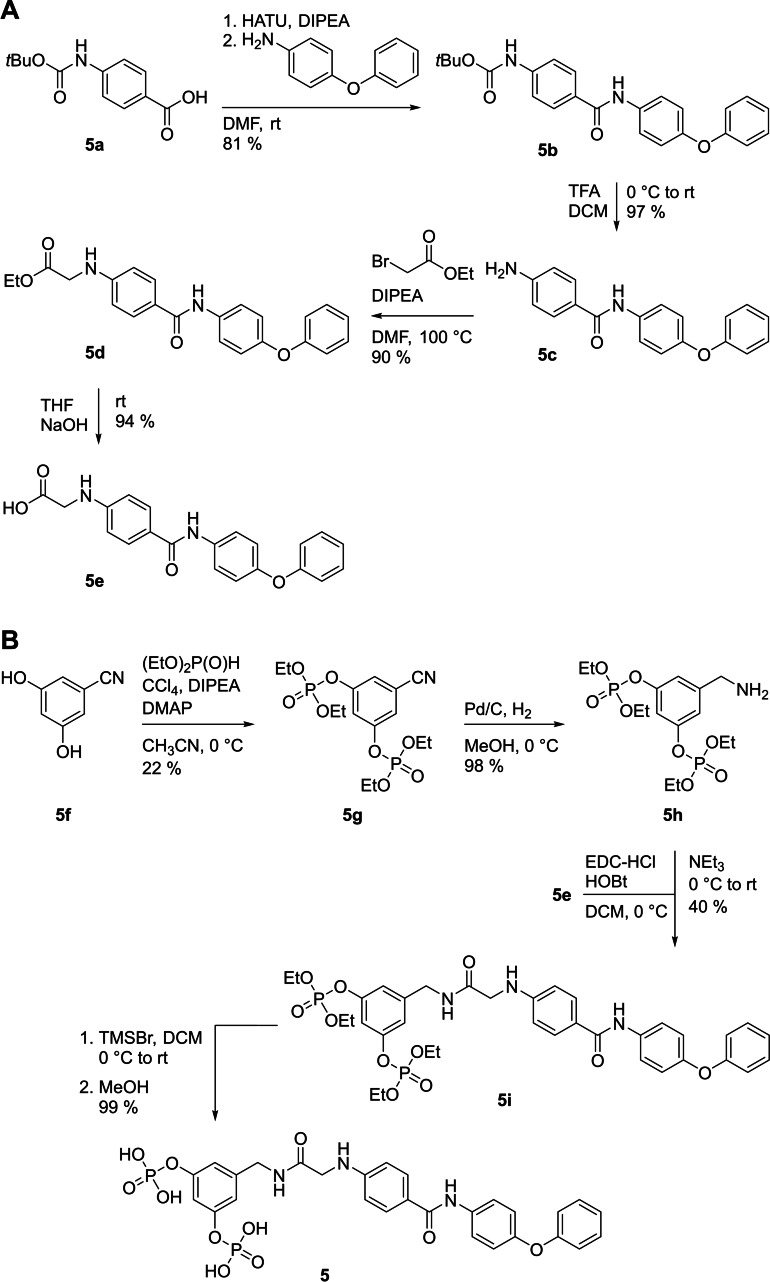
A) Synthesis of the building block **5 e** and B) its use in the synthesis of **5**.

Synthesis of **5** started with coupling of 4‐((*tert*‐butoxycarbonyl)amino)benzoic acid (**5 a**) with 4‐phenoxyaniline to the carbamate **5 b**, which was deprotected to the amine **5 c** (Figure 2A). Reaction with ethyl bromoacetate afforded the ester **5 d**, which was saponificated to provide the acid **5 e** (Figure 2A). In parallel, Atherton‐Todd phosphorylation of 3,5‐dihydroxybenzonitrile (**5 f**, Figure 2B) afforded the nitrile **5 g**. Reduction of the nitrile functionality of **5 g** resulted in formation of the amine **5 h**, which was coupled with the acid **5 e** to give the amide **5 i**. Trimethylsilylbromide‐mediated cleavage of the ester functionalities of **5 i** provided the target compound **5**.

**Figure 3:**
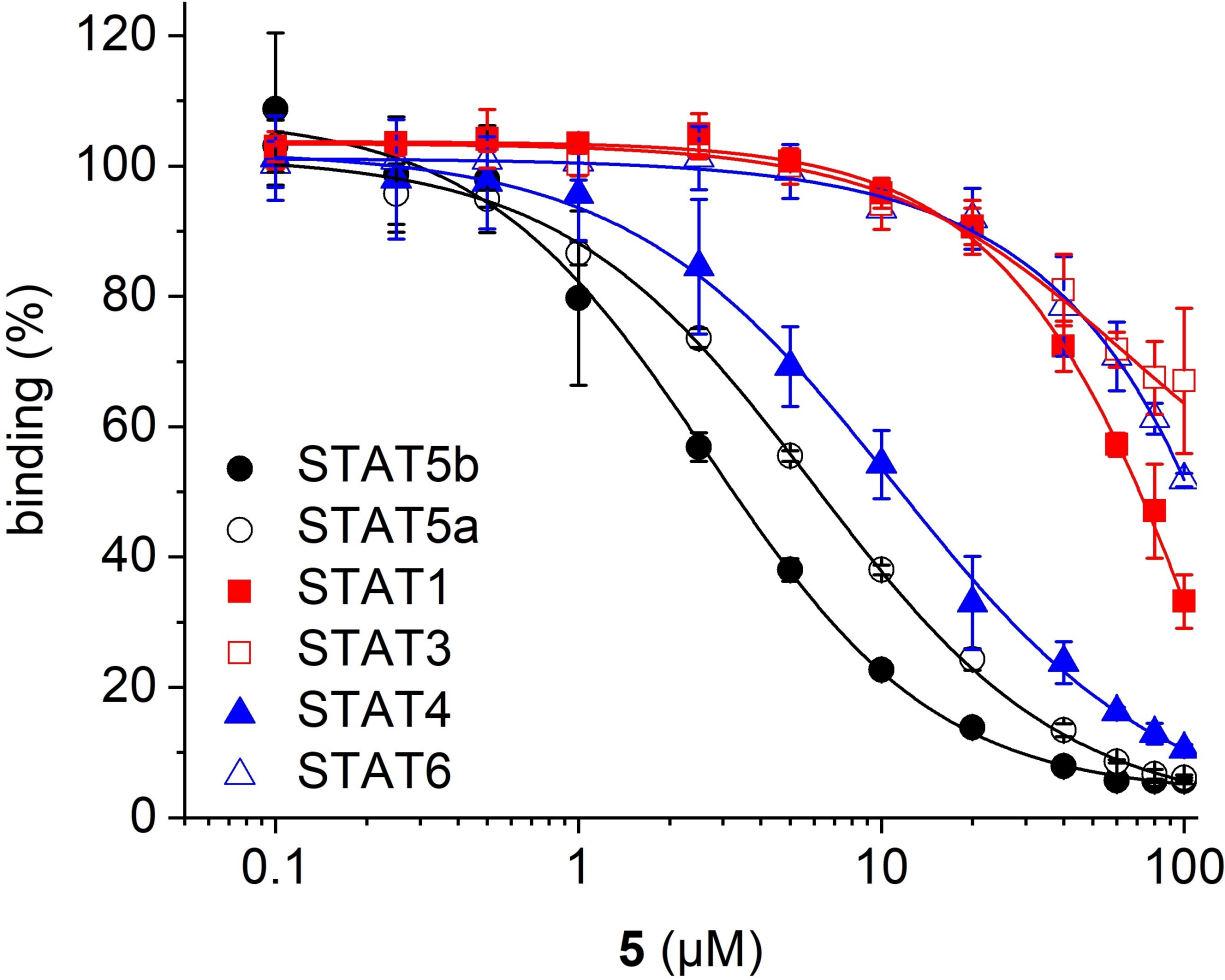
Activity of **5** in fluorescence polarization assays.

The amine **5** showed similar activities against STAT5b (K_i_=1.5±0.1 μM, Tables 1 and S1) and STAT5a (K_i_=3.1±0.1 μM) as **4**. Selectivity analysis revealed that it also inhibits STAT4 (K_i_=5.3±1.3 μM), but has more than tenfold selectivity for STAT5b over STAT1, STAT3, and STAT6 (Figure 3, Table S2).

In parallel studies, we explored the possibility of increasing the STAT5a/5b affinity of resorcinol bisphosphate (**1**) by fluorine substituents.^[14]^ While introduction of a fluorine atom in the 4‐position (compound **6**, Tables 1 and S1) did not improve the activity as compared to resorcinol bisphosphate [K_i_ (STAT5a)=29.4±0.6 μM, K_i_ (STAT5b)=27.4±2.8 μM], a fluorine in the 2‐position (compound **7**) increased the activity by a factor of two to three [K_i_ (STAT5a)=10.8±0.5 μM, K_i_ (STAT5b)=7.6±0.4 μM, Tables 1 and S1] as compared to **1**. This was rationalized by additional polar interactions of the fluorine atom with the side chains of Lys600, and possibly also Arg618 (Figure S2).

**Figure 4:**
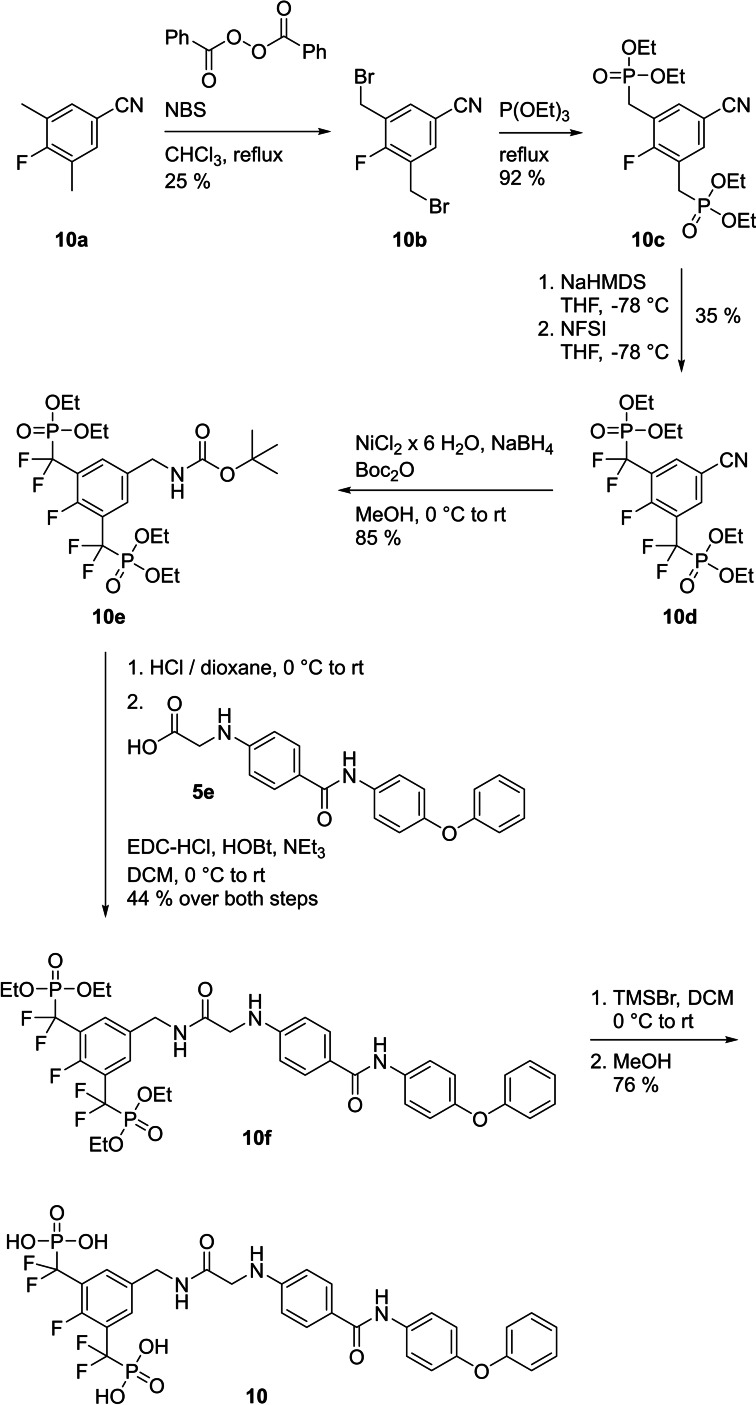
Synthesis of **10**.

Since phosphates are susceptible to hydrolysis by phosphatases, we aimed to convert the structure of **7** into a phosphatase‐stable phosphonate.^[15]^ The bismethylene bisphosphonate **8** was completely inactive against STAT5a/5b (inhibition at 100 μM: 12±3 % against STAT5a, 14±1 % against STAT5b, Tables 1 and S1). In contrast, the bis(difluoromethylene phosphonate) **9** was only slightly less potent than **7** [K_i_ (STAT5a)=15.0±0.6 μM, K_i_ (STAT5b)=18.9±1.5 μM, Tables 1 and S1]. Therefore, **9** was chosen for extension with the hydrophobic building block **5 e** used for synthesis of the bisphosphate **5**. Synthesis of the target compound **10** was achieved in six steps based on 4‐fluoro‐3,5‐dimethylbenzonitrile (**10 a**, Figure 4). Symmetrical monobromination and Arbuzov reaction provided the bisphosphonate **10 c**, which was subjected to deprotonation and electrophilic fluorination with NFSI. The resulting tetrafluorinated nitrile **10 d** was reduced^[16]^ to the *N*‐Boc‐protected amine **10 e**. After removal of the protecting group, coupling with the acid **5 e** generated the amide **10 f**. TMSBr‐mediated cleavage of the phosphonate esters gave the target compound **10**.

**Figure 5:**
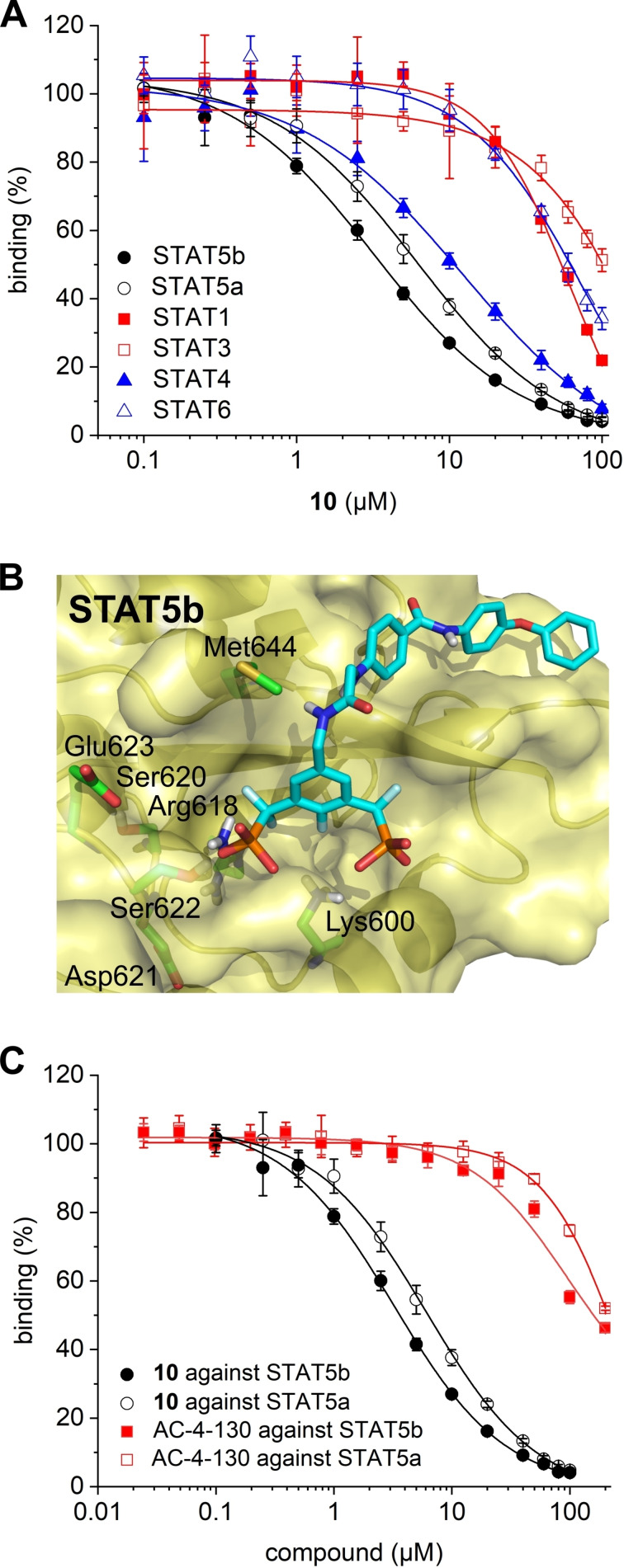
A) Activity of **10** in fluorescence polarization assays against STAT proteins. B) Binding mode of **10** suggested by docking with AutoDockFR. The Figure was created using PyMOL.^[8]^ C) Activity of **10** and AC‐4‐130 against STAT5a and STAT5b in FP assays.

Inhibitor **10** showed single‐digit micromolar activities against both STAT5 proteins [K_i_ (STAT5b)=1.8±0.1 μM, K_i_ (STAT5a)=3.0±0.4 μM, Tables 1 and S1, Figure 5A]. While STAT4 was also inhibited (K_i_=5.3±0.6 μM), selectivity over STAT1, STAT3, and STAT6 was in the order of 10–20‐fold (Figure 5A, Table S3). Compound **10** was dubbed Stafiba (for STAT five b and a inhibitor). Molecular docking of **10** into the STAT5b homology model^[6a]^ suggested a similar binding mode for the more hydrophobic part of the molecule as was postulated for Stafib‐2 (Figure 5B).^[5c]^ We also compared the activity of **10** with that of AC‐4‐130 (Figure S3), a published STAT5 inhibitor.^[3f]^ AC‐4‐130 potently inhibits STAT5 signaling in leukemia cells, but activities in FP assays against the SH2 domains of STAT5a and STAT5b have not been presented.^[3f]^ In our experiments, **10** was substantially more active than AC‐4‐130 in FP assays against the SH2 domains of STAT5a and STAT5b (Figure 5C).

## Conclusion

In conclusion, we have converted resorcinol bisphosphate to Stafiba (**10**), a phosphatase‐stable inhibitor of both STAT5a and STAT5b with single‐digit micromolar activity in fluorescence polarization assays. This involved the extension of resorcinol bisphosphate with building blocks designed to occupy additional subpockets of STAT5a and STAT5b (compounds **4** and **5**), the introduction of a fluorine atom on the aromatic ring between the phosphate groups (compound **7**), and the exchange of the phosphate groups for difluoromethylene phosphonates. Efforts to mask the negative charges of Stafiba for use in cell‐based assays are ongoing and will be reported at a later date.

## Experimental Section

**General information**: ^1^H, ^19^F, ^31^P and ^13^C NMR spectra were recorded on Varian MERCURYplus 400, Bruker Avance III HD and Bruker Fourier 300 spectrometers. The respective solvent peak was used as an internal standard for calibration for ^1^H and ^13^C NMR spectra. ^19^F and ^31^P NMR spectra were referenced using the proton spectrum of the respective compound. Spectra were processed and analyzed using MestReNova Version 14.1.0 software. High resolution mass spectra were recorded using electrospray ionization on an Impact II Bruker Daltonics and a MicrOTOF Bruker Daltonics spectrometer. Infrared spectra were recorded on a Jasco FT/IR‐4100 type A spectrometer. UV/VIS spectra were recorded on a Jasco V‐630 spectrometer. Melting points were measured using a Rapido PHMK apparatus (VEB Wägetechnik) or a melting point M‐560 apparatus (Büchi).

Silica gel 60 M, 0.04–0.2 mm and Geduran Si 60 M, 0.040–0.063 mm from Merck KGaA was used for column chromatography. Pre‐coated TLC sheets ALUGRAM Xtra SIL G/UV254, 0.2 mm, silica gel 60 from Macherey‐Nagel GmbH & Co. KG were used for TLC analyses. Ninhydrin solution (0.30 g ninhydrin, 3.0 mL acetic acid, 100 mL *n*‐butanol), CAM solution (2.5 g phosphomolybdic acid, 1.0 g Cer(IV) sulfate, 6.0 ml concentrated sulfuric acid in 94 mL water), and permanganate solution (3.0 g KMnO_4_, 20 g K_2_CO_3_, 5.0 mL 5 % aqueous NaOH solution in 300 mL water) were used as staining agents. All solvents were distilled prior to use. Anhydrous solvents were purchased from Sigma‐Aldrich. THF was dried over sodium and DCM over CaCl_2_. Deionized water was used. AC‐4‐130 was purchased from Biotrend, Germany, order number AOB36422. ^1^H NMR, ^19^F NMR, and HRMS data were consistent with the published structure.^[3f]^

**Fluorescence polarization assays**: Competitive binding assays based on fluorescence polarization were carried out essentially as previously described.^[4a]^ Unless stated otherwise, final concentrations of the buffer components were: 10 mM Tris (pH 8.0), 50 mM NaCl, 1 mM EDTA, 1 mM DTT, 0.1 % (v/v) Nonidet P‐40 substitute, and 2 % DMSO. Proteins were used at concentrations which reflect the K_d_ values of the interactions between the fluoropeptide and the protein preparations. Protein concentrations used for activity analysis of compound **5** were 91 nM for STAT1, 107 nM for STAT3, 28 nM for STAT4, 122 nM for STAT5a, 56 nM for STAT5b and 101 nM for STAT6. Protein concentrations used for activity analysis of AC‐4‐130 were 103 nM for STAT5a and 62 nM for STAT5b. FP assays for activity analysis of Stafiba (**10**) were carried out in buffer as specified above, but containing 10 % DMSO owing to the limited solubility of **10** in the DMSO stock solution. Proteins were used at the following concentrations, which reflect the approximate K_d_ values of the interactions of fluorescent‐labelled peptide and protein at 10 % DMSO: 42 nM for STAT1, 81 nM for STAT3, 30 nM for STAT4, 185 nM for STAT5a, 99 nM for STAT5b and 75 nM for STAT6. Final concentration of all fluorescent‐labelled peptides was 10 nM. The following peptides were used in assays: 5‐carboxyfluorescein‐GpYDKPHVL for STAT1; 5‐carboxyfluorescein‐GpYLPQTV‐NH_2_ for STAT3; 5‐carboxyfluorescein‐GpYLPQNID for STAT4; 5‐carboxyfluorescein‐GpYLVLDKW for STAT5a/5b, and 5‐carboxyfluorescein‐GpYVPWQDLI for STAT6. Protein and test compounds were incubated for 1 h prior to addition of the fluorescein‐labeled peptide. Fluorescence polarization was measured after another 60 min using an Infinite F500 plate reader (Tecan). IC_50_ values were converted to K_i_‐values using the published equation.^[13]^

## Conflict of interest

T.B. is an inventor on the German patent DE102015201148.8 B4 covering the potential use of phenyl phosphates and their prodrugs as drugs for cancer therapy.

1

## Supporting information

As a service to our authors and readers, this journal provides supporting information supplied by the authors. Such materials are peer reviewed and may be re‐organized for online delivery, but are not copy‐edited or typeset. Technical support issues arising from supporting information (other than missing files) should be addressed to the authors.

Supporting Information

## Data Availability

The data that support the findings of this study are available in the supplementary material of this article.
