# Supplementary material for: Stafiba: A STAT5‐Selective Small‐Molecule Inhibitor
Source: Chembiochem. 2022 Nov 24;24(1):e202200553. doi: 10.1002/cbic.202200553 (PMC10099813; doi:10.1002/cbic.202200553)
Supplement: Supplementary file 1 — Supporting Information [file CBIC-24-0-s001.pdf]

ChemBioChem

Supporting Information

Stafiba: A STAT5-Selective Small-Molecule Inhibitor

Katrin S. Eckhardt, Theresa Münzel, Julian Gräb, and Thorsten Berg*

Table of Contents

Figure S1 2

Figure S2 2

Figure S3 2

Table S1 3

Table S2 4

Table S3 4

Chemical synthesis and spectroscopic characterization 4

Molecular docking 34

NMR spectra 35

Supporting references 40

Figure S1

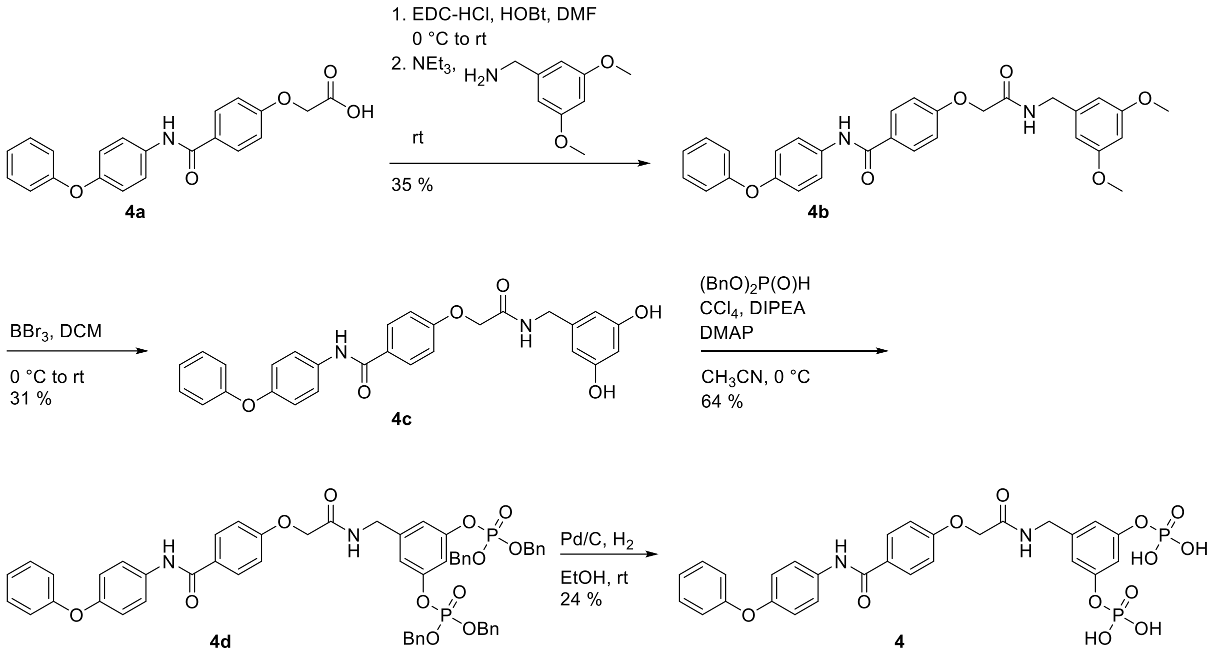

Figure S1: Synthesis of **4**.

Figure S2

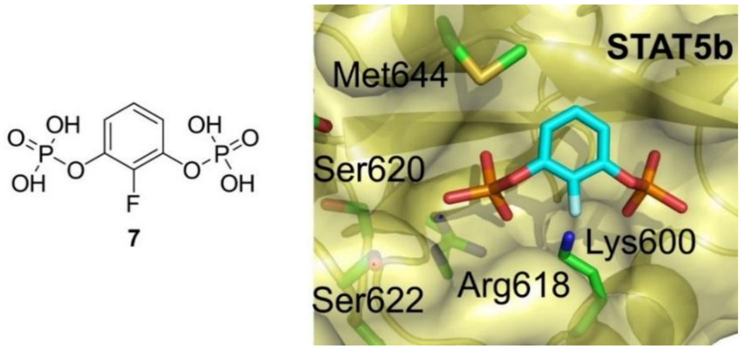

Figure S2: Binding mode of **7** suggested by docking with AutoDockFR.

Figure S3

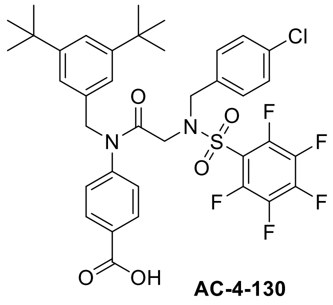

Figure S3: Structure of AC-4-130.[1]

Table S1

No	Structure	IC_{50} (µM) (STAT5a) or maximum inhibition at 100 µM (%)	K_i (µM) (STAT5a)	IC_{50} (µM) (STAT5b) or maximum inhibition at 100 µM (%)	K_i (µM) (STAT5b)
**1**	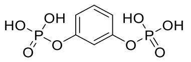	47.2 ± 9.9 µM	23.3 ± 4.9 µM	41.6 ± 1.4 µM	20.4 ± 0.7 µM
**2**	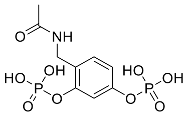	50 ± 3 % inhibition	n/a	85.7 ± 3.2 µM	42.1 ± 1.6 µM
**3**	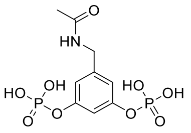	73.8 ± 7.7 µM	36.4 ± 3.8 µM	38.1 ± 1.6 µM	18.7 ± 0.8 µM
**4**	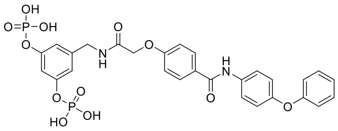	5.2 ± 1.0 µM	2.5 ± 0.5 µM	1.9 ± 0.3 µM	0.9 ± 0.1 µM
**5**	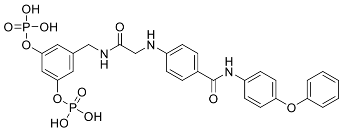	6.4 ± 0.2 µM	3.1 ± 0.1 µM	3.1 ± 0.3 µM	1.5 ± 0.1 µM
**6**	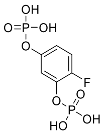	59.4 ± 1.2 µM [a]	29.4 ± 0.6 µM [a]	55.8 ± 5.6 µM	27.4 ± 2.8 µM
**7**	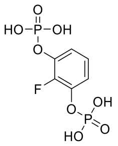	21.8 ± 0.9 µM	10.8 ± 0.5 µM	15.5 ± 0.8 µM	7.6 ± 0.4 µM
**8**	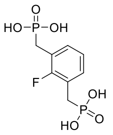	12 ± 3 % inhibition	n/a	14 ± 1 % inhibition	n/a
**9**	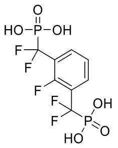	30.7 ± 1.3 µM	15.0 ± 0.6 µM	38.4 ± 3.0 µM	18.9 ± 1.5 µM
**10**	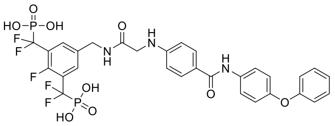	6.2 ± 0.7 µM	3.0 ± 0.4 µM	3.7 ± 0.2 µM	1.8 ± 0.1 µM

Table S1: Activities of synthesized compounds against STAT5a and STAT5b in FP assays. Experiments were carried out in triplicate (n = 3) unless stated otherwise. [a] n=2. n/a: not applicable. K_i values were calculated from IC_{50} values via the published equation.[2]

Table S2

No	Structure	STAT1	STAT3	STAT4	STAT5a	STAT5b	STAT6
**5**	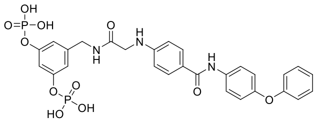	IC_{50} = 70.4 ± 3.1 µM<br><br>K_i = 34.6 ± 1.5 µM	33 ± 11 % inhibition at 100 µM	IC_{50} = 11.3 ± 2.7 µM<br><br>K_i = 5.3 ± 1.3 µM	IC_{50} = 6.4 ± 0.2 µM<br><br>K_i = 3.1 ± 0.1 µM	IC_{50} = 3.1 ± 0.3 µM<br><br>K_i = 1.5 ± 0.1 µM	48 ± 1 % inhibition at 100 µM

Table S2: Activities of **5** against STAT proteins in FP assays.

Table S3

No	Structure	STAT1	STAT3	STAT4	STAT5a	STAT5b	STAT6
**10**	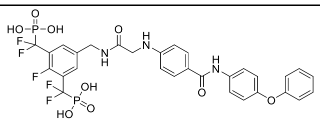	IC_{50} = 53.8 ± 1.6 µM<br><br>K_i = 26.0 ± 0.8 µM	49 ± 3 % inhibition at 100 µM	IC_{50} = 11.2 ± 1.2 µM<br><br>K_i = 5.3 ± 0.6 µM	IC_{50} = 6.2 ± 0.7 µM<br><br>K_i = 3.0 ± 0.4 µM	IC_{50} = 3.7 ± 0.2 µM<br><br>K_i = 1.8 ± 0.1 µM	IC_{50} = 61.3 ± 2.7 µM<br><br>K_i = 30.1 ± 1.3 µM

Table S3: Activities of **10** against STAT proteins in FP assays.

Chemical synthesis and spectroscopic characterization

1,3-Phenylene bis(dihydrogen phosphate) (**1**)

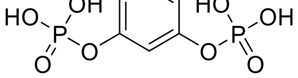

Synthesis of **1** has been described.[3]

N-(2,4-dihydroxybenzyl)acetamide (**2a**)

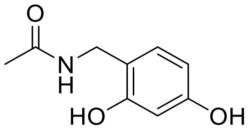

To a solution of acetic acid (0.296 mL, 0.474 mmol, 1.0 eq) in 1.5 mL anhydrous DMF at 0 °C was added EDC-HCl (95.8 mg, 0.502 mmol, 1.0 eq) and HOBt (76.5 mg, 0.500 mmol, 1.0 eq). The solution was allowed to warm to room temperature after 0.5 h and was stirred for another 1.5 h. Triethylamine (0.278 mL, 1.96 mmol, 4.0 eq) and 4-(aminomethyl)benzene-1,3-diol (70 mg, 0.501 mmol, 1.0 eq) were added and the reaction mixture was stirred overnight at room temperature. The solution was neutralized using a 3 M aqueous HCl solution and the aqueous phase extracted with ethyl acetate. The combined organic layers were dried over Na_2SO_4, filtered and evaporated. Purification of the crude product by column chromatography using a gradient of 5 % to 10 % methanol in DCM (*v*/*v*) yielded 51.6 mg (0.285 mmol, 57 %) **2a** as colorless solid.

^{1}H NMR (400 MHz, [D$_6$]DMSO) δ = 9.45 (s, 1H), 9.11 (s, 1H), 8.19 (bs, 1H), 6.86 (d, *J*=8.2 Hz, 1H), 6.24 (d, *J*=2.3 Hz, 1H), 6.16 (dd, *J*=8.2, 2.3 Hz, 1H), 4.04 (d, *J*=5.7 Hz, 2H), 1.83 ppm (s, 3H).

^{13}C NMR (101 MHz, [D$_6$]acetone) δ = 173.0, 159.5, 158.2, 132.9, 117.7, 107.0, 104.8, 40.1, 22.3 ppm.

HRMS (ESI/TOF) m/z: [M - H]$^-$ calcd for $C_9H_{10}NO_3^-$ 180.0666; found: 180.0663.

IR (KBr): ṽ = 3279, 2692, 2631, 2458, 1747, 1624, 1563, 1556, 1538, 1520, 1513, 1462, 1455, 1445, 1376, 1357, 1297, 1231, 1169, 1113, 1045, 1023, 996, 974, 825, 787, 763, 603, 465 cm^{-1}.

UV/Vis (methanol): λ_{max} = 279, 224, 208 nm.

R$_f$ (5 % methanol/DCM, *v*/*v*) = 0.34.

MP: 136 °C.

4-(Acetamidomethyl)-1,3-phenylene tetrabenzyl bis(phosphate) (**2b**)

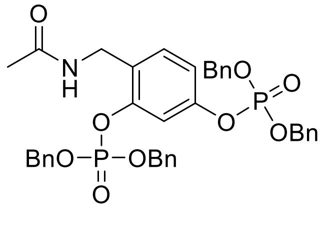

To a solution of **2a** (80.0 mg, 0.442 mmol, 1.0 eq) in 2.5 mL anhydrous acetonitrile was added carbon tetrachloride (0.422 mL, 4.44 mmol, 10 eq), DIPEA (0.308 mL, 1.77 mmol, 4.0 eq.) and a catalytic amount of DMAP. The solution was cooled to 0 °C and dibenzyl phosphite (0.292 mL, 1.32 mmol, 3.0 eq.) was added. The reaction mixture was stirred at 0 °C for 2 h

before being quenched by the addition of 1 mL of a 0.5 M aqueous KH_2PO_4 solution. The organic and aqueous phases were separated and the aqueous phase repeatedly extracted with ethyl acetate. The combined organic layers were dried over Na_2SO_4, filtered and evaporated. The crude product was purified by column chromatography using a gradient of 20 % ethyl acetate in hexanes (*v*/*v*) to 100 % ethyl acetate yielding 115 mg (0.163 mmol, 37 %) **2b** as a colorless solid.

^{1}H NMR (400 MHz, $CDCl_3$) δ = 7.40 – 7.27 (m, 20H), 7.05 (s, 2H), 6.96 (d, *J*=8.0 Hz, 1H), 6.77 (bs, 1H), 5.08 (d, *J*=8.9 Hz, 8H), 4.31 (d, *J*=5.8 Hz, 2H), 1.90 ppm (s, 3H).

^{31}P NMR (162 MHz, $CDCl_3$) δ = -5.54 (1P), -6.45 ppm (1P).

^{13}C NMR (75 MHz, $CDCl_3$) δ = 170.1, 135.3 (d, *J*=7.1 Hz), 135.1 (d, *J*=6.0 Hz), 132.4, 129.1, 128.8, 128.2, 127.6 (d, *J*=6.0 Hz), 117.8 (d, *J*=2.5 Hz), 113.6 (dd, *J*=5.8, 2.6 Hz), 70.8 (d, *J*=6.2 Hz), 70.3 (d, *J*=5.8 Hz), 37.8, 23.3 ppm.

HRMS (ESI/TOF) m/z: $[M + Na]^+$ calcd for $C_{37}H_{37}NO_9P_2Na^+$ 724.1836; found: 724.1850.

IR (KBr): $\tilde{\nu}$ = 3592, 3541, 3465, 3303, 3065, 3034, 2956, 2919, 2849, 1667, 1497, 1273, 994 cm^{-1}.

UV/Vis (acetonitrile): λ_{max} = 259, 210 nm.

R_f (50 % ethyl acetate/hexanes, *v*/*v*) = 0.32.

MP: decomposition at 189 - 192 °C.

4-(Acetamidomethyl)-1,3-phenylene bis(dihydrogen phosphate) (**2**)

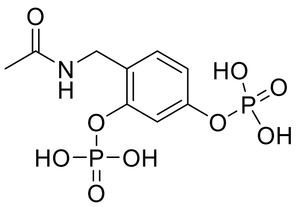

To a solution of **2b** (115 mg, 0.163 mmol, 1.0 eq.) in 25 mL ethanol was added a catalytic amount of Pd/C (10 wt-%, wet) under an Ar atmosphere. The Ar atmosphere was exchanged for H_2 and the reaction mixture stirred for 3 h at room temperature. The mixture was filtered through celite and the solvent was evaporated. The residue was taken up in a few mL of water and the solution was repeatedly washed with DCM. The water was removed by lyophilisation to yield 47.2 mg (0.138 mmol, 86 %) of **2** as a transparent, colorless solid.

^{1}H NMR (400 MHz, D_2O) δ = 7.28 (d, *J*=8.0 Hz, 1H), 7.14 (s, 1H), 6.99 (d, *J*=8.0 Hz, 1H), 4.37 (s, 2H), 2.02 ppm (s, 3H).

^{31}**P NMR** (162 MHz, D_2O) δ = -4.3 ppm.

^{13}**C NMR** (101 MHz, D_2O) δ = 174.0, 151.9 – 151.0 (m), 150.6 – 149.7 (m), 129.9, 124.9 – 124.7, 116.2, 112.5, 38.3, 21.7 ppm.

HRMS (ESI/TOF) m/z: $[M - H]^-$ calcd for $C_9H_{12}NO_9P_2^-$ 339.9993; found: 339.9976.

IR (film): $\tilde{\nu}$ = 3420, 3304, 2919, 2850, 2353, 2256, 1383, 1157, 1112, 1051, 1026, 1008, 601, 456 cm^{-1}.

UV/Vis (D_2O): λ_{max} = 268, 215 nm.

R_f (10 % acetonitrile/water, *v*/*v*) = 0.95.

MP: 70-72 °C.

N-(3, 5-dimethoxybenzyl)acetamide (**3a**)

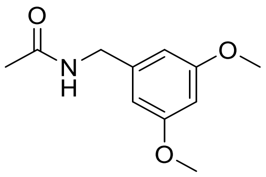

To a solution of (3,5-dimethoxyphenyl)methanamine (400 mg, 2.39 mmol, 1.0 eq) in 12 mL anhydrous DCM was added triethylamine (1.00 mL, 7.20 mmol, 3.0 eq) and acetic anhydride (0.453 mL, 4.81 mmol, 2.0 eq). The reaction mixture was stirred at room temperature for 2 h. Water was added to quench the reaction and the organic and aqueous phases were separated. The aqueous phase was extracted with a total amount of 100 mL DCM and the combined organic layers dried over Na_2SO_4, filtered and evaporated. The crude product was purified by column chromatography using 5 % methanol in DCM (*v*/*v*) to yield 509 mg (2.44 mmol, quant.) of **3a** as a colorless solid.

^{1}**H NMR** (400 MHz, $CDCl_3$) δ = 6.42 (s, 2H), 6.37 (s, 1H), 5.75 (bs, 1H), 4.37 (d, *J* = 5.6 Hz, 2H), 3.78 (s, 6H), 2.03 ppm (s, 3H).

^{13}**C NMR** (101 MHz, $CDCl_3$) δ = 170.0, 161.2, 140.7, 105.9, 99.5, 55.5, 44.0, 23.4 ppm.

HRMS (ESI/TOF) m/z: $[M + Na]^+$ calcd for $C_{11}H_{15}NO_3Na^+$ 232.0944; found: 232.0936.

IR (KBr): $\tilde{\nu}$ = 3397, 3293, 3073, 3000, 2965, 2939, 2888, 2842, 2254, 1631, 1611, 1600, 1545, 1430, 1362, 1209, 1155 cm^{-1}.

UV/Vis (acetonitrile): λ_{max} = 280, 223 nm.

R_f (5 % methanol/DCM, *v*/*v*) = 0.32.

MP: 104 °C, (lit. 105-105.5 °C).[4]

N-(3,5-dihydroxybenzyl)acetamide (**3b**)

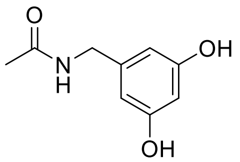

3a (376 mg, 1.75 mmol, 1.0 eq) was dissolved in 6 mL anhydrous DCM. The solution was cooled to 0° C and a 1 M solution of BBr_3 in DCM (8.77 mL, 8.77 mmol, 5.0 eq) was added dropwise. The reaction mixture was stirred at room temperature for 3.5 h. 10 mL of water were added to quench the reaction and the mixture was extracted with a total of 220 mL ethyl acetate. The combined organic layers were dried over Na_2SO_4, filtered and evaporated. The crude product was purified by column chromatography using 10 % methanol in DCM (*v*/*v*) to yield 253 mg (1.41 mmol, 80 %) **3b** as an off-white solid.

^{1}H NMR (400 MHz, [D_6]DMSO) δ 9.13 (s, 2H), 8.21 (bs, 1H), 6.09 (s, 2H), 6.05 (s, 1H) 4.05 (d, *J* = 5.9 Hz, 2H), 1.85 ppm (s, 3H).

^{13}C NMR (101 MHz, [D_6]DMSO) δ = 168.9, 158.3, 141.5, 105.2, 100.9, 39.9, 22.6 ppm.

HRMS (ESI/TOF) m/z: [M - H]$^-$ calcd for $C_9H_{10}NO_3^-$ 180.0666; found 180.0662.

IR (KBr): $\tilde{\nu}$ = 3326, 3215, 3107, 2996, 2676, 1649, 1611, 1306, 1165, 701 cm^{-1}.

UV/Vis (DMSO): λ_{max} = 280, 209 nm.

R_f (10 % methanol/DCM, *v*/*v*) = 0.34.

MP: 164 °C.

5-(Acetamidomethyl)-1,3-phenylene tetrabenzyl bis(phosphate) (**3c**)

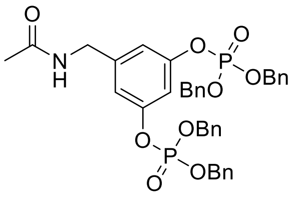

To a suspension of **3b** (50.0 mg, 0.276 mmol, 1.0 eq) in 1.5 mL anhydrous acetonitrile was added CCl_4 (0.250 mL, 2.76 mmol, 10 eq), DIPEA (0.191 mL, 1.10 mmol, 4.0 eq) and a catalytic amount of DMAP. The suspension was cooled to 0 °C and dibenzyl phosphite

(0.140 mL, 0.828 mmol, 3.0 eq) was added dropwise. The yellow solution was stirred at 0 °C for 3.5 h. To quench the reaction, 0.7 mL of an aqueous 0.5 M KH_2PO_4 solution were added. The mixture was extracted six times with ethyl acetate and three times with DCM. The combined organic extracts were dried over anhydrous $MgSO_4$, filtered and the solvent evaporated under reduced pressure. The crude product was purified by column chromatography using 10 % methanol in DCM (*v/v*). Purification yielded 80.0 mg **3c** (0.113 mmol, 41 %) as a colorless solid.

^{1}H NMR (400 MHz, $CDCl_3$) δ = 7.32 (s, 20H), 6.92 – 6.83 (m, 3H), 5.61 (bs, 1H), 5.09 (d, *J*=8.6 Hz, 8H), 4.27 (d, *J*=5.9 Hz, 2H), 1.97 ppm (s, 3H).

^{13}C NMR (101 MHz, $CDCl_3$) δ = 151.7, 135.4 (d, *J*=6.7 Hz), 128.9, 128.8, 128.3, 116.1 (d, *J*=4.2 Hz), 111.8 (d, *J*=4.3 Hz), 70.3 (d, *J*=5.6 Hz), 43.0, 23.6 ppm.

HRMS (ESI/TOF) m/z: $[M + Na]^+$ calcd for $C_{37}H_{37}NO_9P_2Na^+$ 724.1836; found: 724.1868.

R_f (10 % methanol/DCM, *v/v*) = 0.53.

5-(Acetamidomethyl)-1,3-phenylene bis(dihydrogen phosphate) (**3**)

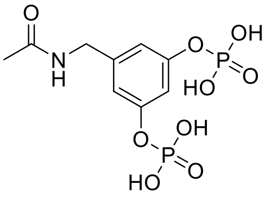

3c (72.5 mg, 0.103 mmol, 1.0 eq) was dissolved in 25 mL ethanol and Pd/C (10 wt-%, wet) was added under an Ar atmosphere. Argon was exchanged for H_2 and the reaction mixture stirred at room temperature for 3 h. The mixture was filtered through celite and the solvent removed under reduced pressure. The residue was taken up in 5 mL water and was washed two times with 60 mL DCM. Water was removed by lyophilisation yielding 30.6 mg **3** (0.0897 mmol, 87 %) as a colorless solid.

^{1}H NMR (400 MHz, D_2O) δ = 6.9 (s, 3H), 4.4 (s, 2H), 2.1 ppm (s, 3H).

^{31}P NMR (162 MHz, D_2O) δ = -4.2 ppm.

R_f (10 % acetonitrile/water, *v/v*) = 0.95.

2-(4-((3-Phenoxyphenyl)carbamoyl)phenoxy) acetic acid (**4a**)

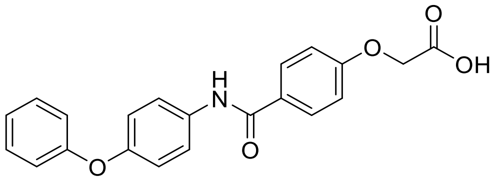

Synthesis of **4a** has been described.[5]

4-(2-((3,5-Dimethoxybenzyl)amino)-2-oxoethoxy)-*N*-(4-phenoxyphenyl)benzamide (**4b**)

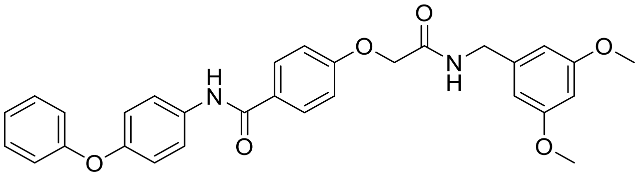

To a solution of acid **4a** (190 mg, 0.523 mmol, 0.44 eq) in 4 mL anhydrous DMF at 0 °C was added EDC-HCl (217 mg, 1.13 mmol, 1.0 eq) and HOBt (207 mg, 1.13 mmol, 1.0 eq). The suspension was stirred at 0 °C for 30 min, allowed to warm to room temperature. The reaction mixture was stirred for 1.5 h before the addition of 5-(aminomethyl)benzene-1,3-diol (189 mg, 1.13 mmol, 1.0 eq) and triethylamine (0.633 mL, 4.54 mmol, 4.0 eq) and then stirred for 19.5 h at room temperature. Water was added and the solution was acidified by addition of 3 M aqueous HCl solution (pH = 1). The precipitate was filtered off and washed with cold water yielding 200 mg (0.391 mmol, 35 %) **4b** as an off-white solid.

^{1}H NMR (400 MHz, $CDCl_3$) δ = 7.86 (d, *J*=8.3 Hz, 2H), 7.59 (d, *J*=8.8 Hz, 2H), 7.33 (t, *J*=8.1 Hz, 2H), 7.09 (t, *J*=8.1 Hz, 2H) , 6.98-7.05 (m, 6H), 6.81 (bs, 1H), 6.44 – 6.40 (m, 2H), 6.40 – 6.36 (m, 1H), 4.60 (s, 2H), 4.49 (d, *J*=6.0 Hz, 2H), 3.77 ppm (s, 6H).

^{13}C NMR (101 MHz, $CDCl_3$) δ = 167.6, 165.0, 161.3, 159.3, 157.6, 153.9, 140.0, 133.5, 129.9, 129.3, 128.9, 123.3, 122.1, 120.4, 118.7, 114.8, 105.9, 99.2, 66.7, 55.5, 43.3 ppm.

HRMS (ESI/TOF) m/z: $[M + Na]^+$ calcd for $C_{37}H_{37}NNaO_9P_2^+$ 535.1840; found: 535.1828.

IR (KBr): $\tilde{\nu}$ = 3424, 3312, 3065, 3000, 2928, 2839, 1754, 1668, 1607, 1537, 1508, 1488, 1223, 845 cm^{-1}.

UV/Vis (chloroform): λ_{max} = 277, 243 nm.

R_f (50 % acetonitrile/water, *v*/*v*) = 0.46.

MP: 176 °C.

4-(2-((3,5-Dihydroxybenzyl)amino)-2-oxoethoxy)-*N*-(4-phenoxyphenyl)benzamide (**4c**)

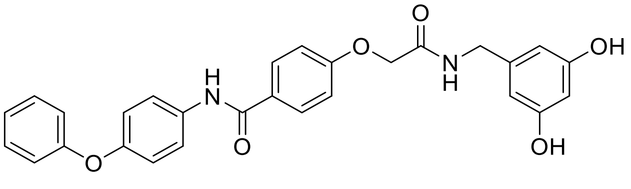

To a suspension of **4b** (100 mg, 0.195 mmol, 1.0 eq) in 3 mL anhydrous DCM at 0 °C was added a 1 M BBr_3 solution in DCM (0.976 mL, 0.976 mmol, 5.0 eq) and the reaction mixture was stirred at room temperature for 3.5 h. The mixture was quenched with water and was then extracted ten times with ethyl acetate. The combined organic extracts were dried over anhydrous Na_2SO_4, filtered and the solvent evaporated *in vacuo*. The crude product was purified by column chromatography using 10 % methanol in DCM (*v*/*v*) to obtain 29.2 mg (0.0602 mmol, 31 %) **4c** as a colorless solid.

^{1}H NMR (400 MHz, CD_3OD) δ = 7.94 (d, *J*=8.8 Hz, 2H), 7.65 (d, *J*=8.4 Hz, 2H), 7.34 (t, *J*=8.0 Hz, 2H), 7.14 – 7.08 (m, 3H), 7.02 – 6.96 (m, 4H), 6.25 – 6.23 (m, 2H), 6.17 – 6.15 (m, 1H), 4.65 (s, 2H), 4.33 ppm (s, 2H).

^{13}C NMR (101 MHz, CD_3OD) δ = 170.5, 168.1, 162.0, 159.7, 159.0, 155.2, 141.9, 135.4, 130.8, 130.6, 129.3, 124.2, 124.1, 120.2, 119.5, 115.7, 106.8, 102.4, 68.2, 43.7 ppm.

HRMS (ESI/TOF) m/z: $[M + Na]^+$ calcd for $C_{28}H_{24}N_2O_6Na^+$ 507.1527; found: 507.1537.

IR (KBr): $\tilde{\nu}$ = 3420, 3312, 2922, 2853, 2659, 2615, 2070, 2031, 1892, 1657, 1605, 1507, 1488, 1249, 1222, 1162 cm^{-1}.

UV/Vis (methanol): λ_{max} = 276, 209 nm.

R_f (10 % methanol/DCM, *v*/*v*) = 0.51.

MP: 194 °C.

Tetrabenzyl (5-((2-(4-((4-phenoxyphenyl)carbamoyl)phenoxy)acetamido)methyl)-1,3-phenylene) bis(phosphate) (**4d**)

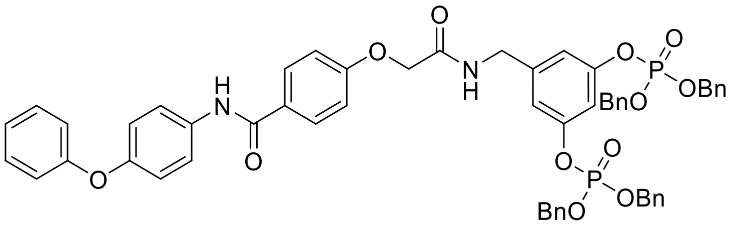

To a suspension of **4c** (43.0 mg, 0.0887 mmol, 1.0 eq) in 1.5 mL anhydrous acetonitrile was added carbon tetrachloride (0.0845 mL, 0.887 mmol, 10 eq), DIPEA (0.0613 mL, 0.352 mmol, 4.0 eq.) and a catalytic amount of DMAP. The suspension was cooled to 0 °C and dibenzyl phosphite (0.0453 mL, 0.266 mmol, 3.0 eq.) was added dropwise. The reaction mixture was stirred at 0 °C for 30 min before being quenched by the addition of 1 mL of a 0.5 M aqueous KH_2PO_4 solution. The organic and aqueous phases were separated and the aqueous phase repeatedly extracted with ethyl acetate. The combined organic layers were dried over Na_2SO_4, filtered and evaporated. The crude product was purified by column chromatography using 3 % methanol in DCM (*v*/*v*) yielding 57.0 mg (0.0567 mmol, 64 %) **4d** as a colorless oil.

^{1}H NMR (400 MHz, CD_3OD) δ = 7.93 (d, *J*=8.9 Hz, 1H), 7.64 (d, *J*=8.5 Hz, 2H), 7.39 – 7.26 (m, 22H), 7.14 – 7.04 (m, 3H), 7.03 – 6.94 (m, 4H), 6.92 (s, 2H), 6.84 (s, 1H), 5.10 (d, *J*=9.2 Hz, 8H), 4.63 (s, 2H), 4.37 ppm (s, 2H).

^{31}P NMR (162 MHz, CD_3OD) δ = -7.69 ppm.

^{13}C NMR (101 MHz, CD_3OD) δ = 169.4, 156.5, 153.7, 150.8 (d, *J*=7.1 Hz), 142.4, 135.2, 134.0, 129.4, 129.3, 128.6 – 127.8 (m), 127.6-127.2 (m), 123.0-122.6 (m), 118.8, 118.1, 115.7, 114.3, 110.9, 70.3 (d, *J*=5.9 Hz), 66.8, 41.5 ppm (d, *J*=14.4 Hz).

HRMS (ESI/TOF) m/z: $[M + H]^+$ calcd for $C_{56}H_{51}N_2O_{12}P_2^+$ 1005.2912; found: 1005.2960.

IR (film): $\tilde{\nu}$ = 3426, 3307, 3089, 3065, 3034, 3009, 2956, 2896, 2853, 2359, 2339, 1957, 1888, 1768, 1666, 1507, 1221, 1014, 750 cm^{-1}.

UV/Vis (DCM): λ_{max} = 273 nm.

R_f (3 % methanol/DCM, *v*/*v*) = 0.26.

5-((2-(4-((4-Phenoxyphenyl)carbamoyl)phenoxy)acetamido)methyl)-1,3-phenylene bis(dihydrogen phosphate) (**4**)

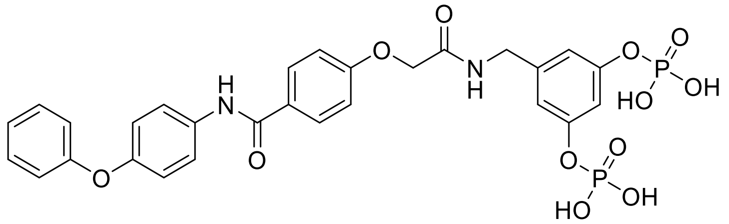

To a solution of **4d** (15.0 mg, 0.015 mmol, 1.0 eq.) in 25 mL ethanol was added a catalytic amount of Pd/C (10 wt-%, wet) under an Ar atmosphere. The Ar atmosphere was exchanged for H_2 and the reaction mixture stirred for 2 h 50 min at room temperature. The mixture was filtered through celite and the solvent was evaporated. Purification of the residue by two

successive column chromatographies using 10 % acetonitrile in water (*v*/*v*) afforded 2.3 mg (0.00357 mmol, 24 %) **4** as a colorless solid.

^{1}H NMR (400 MHz, D_2O) δ = 7.91 (d, *J*=8.6 Hz, 2H), 7.53 (d, *J*=8.5 Hz, 2H), 7.46 (t, *J*=7.9 Hz, 2H), 7.23 (t , *J*=7.9 Hz, 1H), 7.19 – 7.10 (m, 6H), 6.88 (s, 2H), 6.85 (s, 1H), 4.45 ppm (s, 2H).

^{31}P NMR (162 MHz, D_2O) δ = 0.08 ppm.

^{13}C NMR (101 MHz, D_2O) δ = 170.8, 160.3, 156.9, 154.6 (d, *J*=5.9 Hz), 154.4 – 154.0 (m), 54.2, 138.7, 132.6, 130.1, 129.6, 127.1, 124.8, 123.8, 119.4, 118.7, 114.7, 113.4, 111.6, 110.2, 66.6, 42.7 ppm.

HRMS (ESI/TOF) m/z: $[M - H]^-$ calcd for $C_{28}H_{25}N_2O_{12}P_2^-$ 643.0888; found: 643.0876.

IR (KBr): $\tilde{\nu}$ = 3442, 2922, 2857, 1631, 1384, 1155, 1042, 896, 625, 407 cm^{-1}.

UV/Vis (methanol): λ_{max} = 276, 209 nm.

R_f (10 % acetonitrile/water, *v*/*v*) = 0.77.

MP: decomposition at 153 °C.

Tert-butyl (4-((4-phenoxyphenyl)carbamoyl)phenyl)carbamate (**5b**)

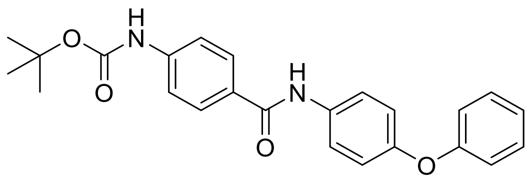

HATU (352 mg, 0.927 mmol, 1.1 eq) and DIPEA (0.484 mL, 2.78 mmol, 3.0 eq) were added to a solution of 4-((*tert*-butoxycarbonyl)amino)benzoic acid (**5a**, 200 mg, 0.842 mmol, 1.0 eq) in 8.5 mL anhydrous DMF. After stirring the mixture for 30 min at room temperature, 4-phenoxyaniline (156 mg, 0.842 mmol, 1.0 eq) was added and the reaction mixture was left to stir overnight. Addition of 20 mL water and filtration of the resulting suspension yielded 275 mg (0.682 mmol, 81 %) **5b** as an off-white solid which was used in subsequent reactions without further purification.

^{1}H NMR (300 MHz, [D_6]DMSO) δ = 10.09 (s, 1H), 9.66 (s, 1H), 7.88 (d, *J*=8.8 Hz, 2H), 7.76 (d, *J*=9.0 Hz, 2H), 7.58 (d, *J*=8.8 Hz, 2H), 7.43 – 7.32 (m, 2H), 7.17 – 7.07 (m, 1H), 7.06 – 6.95 (m, 4H), 1.49 ppm (s, 9H; $(CH_3)_3$).

^{13}C NMR (75 MHz, [D_6]DMSO) δ = 165.0, 157.4 , 152.8, 152.1, 142.8, 135.2, 130.1, 128.7, 128.1, 123.2, 122.3, 119.4, 118.1, 117.3, 79.8, 28.2 ppm.

HRMS (ESI/TOF) m/z: $[M + H]^+$ calcd for $C_{24}H_{25}N_2O_4^+$ 405.1809; found: 405.1824.

IR (KBr): $\tilde{\nu}$ =3647, 3427, 2979, 2930, 1731, 1707, 1645, 1608, 1590, 1509, 1226, 1159 cm^{-1}.

UV/Vis (DCM): λ_{max} = 285, 224 nm.

$\mathbf{R_f}$ (1 % methanol/DCM, *v*/*v*) = 0.27.

MP: 223 °C.

4-Amino-*N*-(4-phenoxyphenyl)benzamide (**5c**)

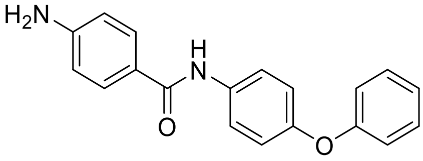

A suspension of 4.75 g (11.7 mmol, 1.0 eq) **5b** in DCM was cooled to 0 °C and kept at this temperature for 5 min before 11.7 mL TFA (152 mmol, 13 eq) were added. The clear solution was allowed to warm to room temperature and stirred until full conversion was observed on TLC. After cooling to 0 °C, the pH of the reaction mixture was adjusted to pH ~ 8 and DCM was removed *in vacuo*. The resulting precipitate was filtered off, washed with cold water and dried affording 3.48 g (11.4 mmol, 97 %) **5c** as an off-white solid.

^{1}H NMR (300 MHz, CD_3OD) δ = 7.73 (d, *J*=8.7 Hz, 2H), 7.62 (d, *J*=8.7 Hz, 2H), 7.33 (t, *J*=7.9 Hz, 2H), 7.08 (t, *J*=7.9 Hz, 1H), 6.97 (d, *J*=8.7 Hz, 4H), 6.71 ppm (d, *J*=8.7 Hz, 2H).

^{13}C NMR (101 MHz, $[D_6]$DMSO) δ = 159.5, 149.7, 145.4, 144.0, 126.3, 121.3, 120.9, 114.6, 113.9, 110.8, 109.9, 105.2 ppm.

HRMS (ESI/TOF) m/z: $[M + Na]^+$ calcd for $C_{19}H_{16}N_2O_2Na^+$ 327.1104; found: 327.1119.

IR (KBr): $\tilde{\nu}$ = 3312, 3065, 2931, 2870, 2587, 1746, 1703, 1666, 1608, 1508, 1488, 1224 cm^{-1}.

UV/Vis (DCM): λ_{max} = 292, 230 nm.

$\mathbf{R_f}$ (5 % methanol/DCM, *v*/*v*) = 0.27.

MP: 132 °C.

Ethyl (4-((4-phenoxyphenyl)carbamoyl)phenyl)glycinate (**5d**)

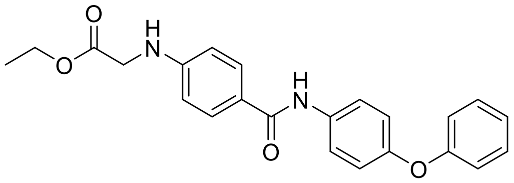

To 1.93 g **5c** (6.33 mmol, 1.0 eq) dissolved in anhydrous DMF was added 2.21 mL (12.7 mmol, 2.0 eq) DIPEA and 1.05 mL (9.50 mmol, 1.5 eq) ethyl bromoacetate. The resulting solution was stirred at 100 °C overnight. After cooling to room temperature, the pH was adjusted to pH ~ 2 and the reaction mixture was poured onto ice. After 30 mins the resulting precipitate was filtered off, washed with cold water and dried. Purification of the crude product by column chromatography using a gradient of 30 % acetone in hexanes (*v*/*v*) to 100 % acetone yielded 2.22 g (5.70 mmol, 90 %) **5d** as an off-white solid.

^{1}H NMR (400 MHz, $CDCl_3$) δ = 8.01 (s, 1H), 7.75 (d, *J*=8.7 Hz, 2H), 7.70 (bs, 1H), 7.57 (d, *J*=8.7 Hz, 2H), 7.32 (t, *J*=7.5 Hz, 2H), 7.08 (t, *J*=7.5 Hz, 1H), 7.05 – 6.96 (m, 4H); 6.63 (d, *J*=8.7 Hz, 2H), 4.27 (q, *J*=7.0 Hz, 2H; CH_2CH_3), 3.96 (s, 2H; CH_2), 1.32 ppm (t, *J*=7.0 Hz, 3H; CH_2CH_3).

^{13}C NMR (101 MHz, $CDCl_3$) δ = 170.6, 165.5, 157.8, 153.4, 150.0, 134.0, 129.8, 129.0, 123.8, 123.1, 122.0, 119.9, 118.5, 112.4, 61.8, 45.4, 14.3 ppm.

HRMS (ESI/TOF) m/z: $[M + H]^+$ calcd for $C_{23}H_{23}N_2O_4^+$ 391.1652; found: 391.1655.

IR (KBr): $\tilde{\nu}$ = 3647, 3383, 1728, 1644, 1608, 1509, 1488, 1226, 1186, 838 cm^{-1}.

UV/Vis (DCM): λ_{max} = 301, 229, 216 nm.

R_f (30 % acetone/hexanes, *v*/*v*) = 0.33.

MP: 174 °C.

(4-((4-Phenoxyphenyl)carbamoyl)phenyl)glycine (**5e**)

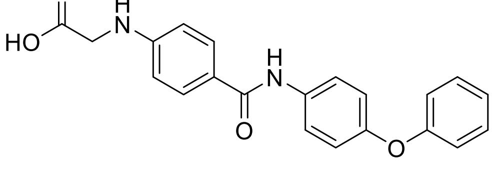

To a solution of 1.12 g ethyl ester **5d** (2.88 mmol, 1.0 eq) in 15 mL THF was added 15 mL of an aqueous 1 M NaOH solution and the resulting mixture was stirred for 1 h at room temperature. After completion of the reaction as observed by TLC, THF was removed under

reduced pressure. The remaining aqueous solution was acidified to pH = 1 and extracted three times with ethyl acetate. The combined organic phases were dried over Na_2SO_4, filtered and evaporated to afford 0.980 g (2.71 mmol, 94 %) **5e** as a colorless solid.

^{1}H NMR (300 MHz, CD_3OD) δ = 7.79 (d, *J*=8.9 Hz, 2H), 7.60 (d, *J*=8.9 Hz, 2H), 7.40 – 7.27 (m, 2H), 7.08 (tt, *J*=7.5, 2.0 Hz, 1H), 7.03 – 6.92 (m, 4H), 6.68 (dt, *J*=9.1, 2.0 Hz, 2H), 3.96 ppm (s, 2H; CH_2).

^{13}C NMR (101 MHz, CD_3OD) δ = 174.4, 168.8, 159.1, 154.9, 152.8, 135.8, 130.8, 130.3, 124.12, 124.07, 123.5, 120.3, 119.4, 112.7, 45.5 ppm (CH_2).

HRMS (ESI/TOF) m/z: $[M - H]^-$ calcd for $C_{21}H_{17}N_2O_4^-$ 361.1194; found: 361.1203.

IR (KBr): $\tilde{\nu}$ = 3399, 3059, 3039, 2912, 2874, 1709, 1637, 1608, 1508, 1488, 1437, 1406, 1319, 1244, 1225, 1187, 835 cm^{-1}.

UV/Vis (methanol): λ_{max} = 294, 206 nm.

R_f (1 % acetic acid/4 % methanol/DCM, *v*/*v*/*v*) = 0.24.

MP: 126 °C.

5-Cyano-1,3-phenylene tetraethyl bis(phosphate) (**5g**)

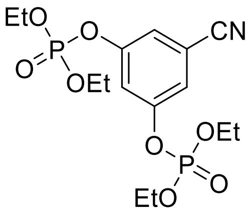

To a solution of 3,5-dihydroxybenzonitrile (**5f**, 200 mg, 1.48 mmol, 1.0 eq) in 11.8 mL dry acetonitrile was added CCl_4 (3.60 mL, 37.3 mmol, 25 eq), DIPEA (0.581 mL, 3.42 mmol, 2.3 eq) and catalytic amounts of DMAP. At 0 °C, diethyl phosphite (0.657 mL, 5.09 mmol, 3.4 eq) was added, and the mixture was stirred for 30 min. Upon completion of the reaction, 30 mL 0.5 M KH_2PO_4 were added. The reaction mixture was repeatedly extracted with a total amount of 60 mL ethyl acetate, dried over Na_2SO_4, filtered and concentrated under reduced pressure to afford a yellow oil. The crude product was purified *via* column chromatography using 60 % ethyl acetate in hexanes (*v*/*v*) followed by basic extraction with an aqueous 0.1 M NaOH solution to give **5g** as a colorless oil (132 mg, 0.324 mmol, 22 %).

^{1}H-NMR (400 MHz, $CDCl_3$): δ = 7.41-7.38 (m, 1H), 7.38-7.36 (m, 2H), 4.30-4.19 (m, 8H), 1.38 ppm (td, *J*=7.1, 1.1 Hz, 12H).

^{31}P-NMR (162 MHz, $CDCl_3$): δ = -6.94 ppm.

^{13}C-NMR (75 MHz, $CDCl_3$): δ = 152.0 (d, *J*=6.3 Hz), 120.3 (d, *J*=5.1 Hz), 117.6 (t, *J*=5.5 Hz), 117.1 , 114.3 , 64.3 (d, *J*=6.3 Hz), 16.2 ppm (d, *J*=6.5 Hz).

HRMS (ESI/TOF) m/z: $[M+H]^+$ calcd for$C_{15}H_{23}NO_8P_2H^+$ 408.0972; found: 408.0985.

IR (film): $\tilde{\nu}$ = 3485, 2989, 2236, 1592, 1439, 1279, 1155, 1029, 983, 830, 754 cm^{-1}.

UV/Vis (DCM): λ_{max} = 282, 230 nm.

R_f (3 % methanol/DCM, *v*/*v*) = 0.15.

5-(Aminomethyl)-1,3-phenylene tetraethyl bis(phosphate) (**5h**)

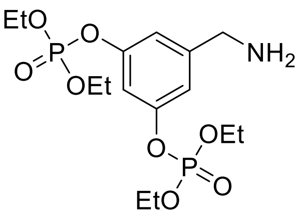

To a solution of **5g** (139 mg, 0.341 mmol, 1.0 eq) in 50 mL methanol were added catalytic amounts of Pd/C. The flask was cooled to 0 °C and N_2 was exchanged for H_2. The mixture was stirred at 0 °C for 1 h, followed by filtration. The solvent was removed under reduced pressure to yield **5h** as a colorless liquid (138 mg, 0.335 mmol, 98 %). The crude product was used in subsequent reactions without further purification.

^{1}H-NMR (400 MHz, $[D_6]$acetone): δ = 7.14 (s, 2H), 7.04 (s, 1H), 4,43 (s, 2H), 4.29-4.14 (m, 8H), 1.33 ppm (td, *J*=7.1, 1.0 Hz, 12H).

HRMS (ESI/TOF) m/z: $[M+Na]^+$ calcd for $C_{15}H_{27}NO_8P_2Na^+$ 434.1104; found: 434.1122.

R_f (60 % acetone/hexanes, *v*/*v*) = 0.63.

3-((Diethoxyphosphoryl)oxy)-5-((2-((4-((4-phenoxyphenyl)carbamoyl)phenyl) amino)acetamido)methyl)phenyl dihydrogen phosphate (**5i**)

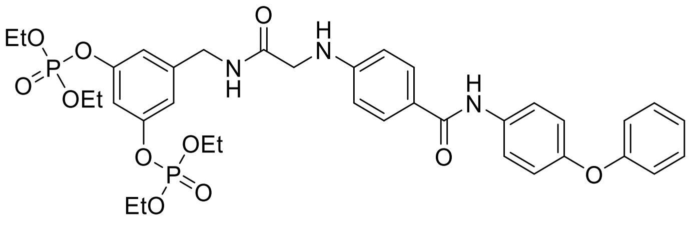

To a solution of **5e** (123 mg, 0.340 mmol, 1.0 eq) in 3.4 mL anhydrous DCM at 0 °C was added EDC-HCl (65.2 mg, 0.340 mmol, 1.0 eq) and HOBt (46.0 mg, 0.340 mmol, 1.0 eq). The

mixture was stirred for 5 min at 0 °C. After that time, triethylamine (0.141 mL, 1.02 mmol, 3.0 eq) and amine **5h** (138 mg, 0.340 mmol, 1.0 eq) were added. The mixture was stirred overnight under N_2 at room temperature. After the addition of 8 mL water and repeated extraction with a total amount of 30 mL ethyl acetate, the combined organic phases were washed with 40 mL brine, dried over Na_2SO_4, filtered and the solvent was removed under reduced pressure. The crude product was purified *via* column chromatography using 5 % methanol in DCM (*v*/*v*) to yield **5i** as a colorless solid (104 mg, 0.149 mmol, 40 %).

^{1}H-NMR (400 MHz, $CDCl_3$): δ = 9.14 (s, 1H), 7.81-7.70 (m, 4H), 7.52 (t, *J* = 6.3 Hz, 1H), 7.34-7.29 (m, 2H), 7.07 (tt, *J*=7.5, 1.1 Hz, 1H), 7.03-6.95 (m, 5H), 6.92-6.90 (m, 2H), 6.45 (d, *J*=8.9 Hz, 2H), 5.37 (t, *J*=5.6 Hz, 1H), 4.36 (d, *J*=6.3 Hz, 2H), 4.23-4.09 (m, 8H), 3.73 (d, *J*=5.5 Hz, 2H), 1.32 ppm (td, *J*=7.1, 1.1 Hz, 12H).

^{31}P-NMR (162 MHz, $CDCl_3$): δ = -7.15 ppm (p, *J*=8.3 Hz).

^{13}C-NMR (101 MHz, $CDCl_3$): δ = 170.7, 166.2, 157.9, 152.8, 151.3 (d, *J*=6.9 Hz), 150.1, 142.1, 134.9, 129.7, 129.4, 124.4, 122.9, 122.1, 119.5, 118.2, 115.8 (d, *J*=5.1 Hz), 112.1, 111.1, 65.0 (d, *J*=6.3 Hz), 47.7, 42.4, 16.1 ppm (d, *J*=6.6 Hz).

HRMS (ESI/TOF) m/z: $[M+Na]^+$ calcd for $C_{36}H_{43}N_3O_{11}P_2Na^+$: 778.2265; found: 778.2260.

IR (KBr): $\tilde{\nu}$ = 3323, 2985, 1661, 1608, 1507, 1489, 1267, 1225, 1030 cm^{-1}.

UV/Vis (methanol): λ_{max} = 299, 214 nm.

R_f (5 % methanol/DCM, *v*/*v*) = 0.21.

MP: 71 °C.

5-((2-((4-((4-Phenoxyphenyl)carbamoyl)phenyl)amino)acetamido)methyl)-1,3-phenylene bis(dihydrogen phosphate) (**5**)

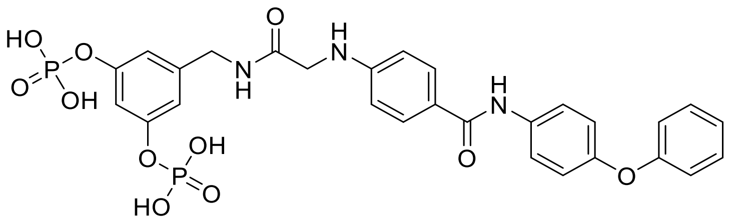

At 0 °C, to a solution of **5i** (20.0 mg, 0.0265 mmol, 1.0 eq) in 1 mL anhydrous DCM was added TMSBr (0.0250 mL, 0.185 mmol, 7.0 eq) dropwise. The mixture was stirred for 43 h under N_2 at room temperature. After that time, 5 mL methanol were added and the solution was evaporated under reduced pressure. The residue was washed with methanol and DCM. The

crude product was purified *via* reversed-phase column chromatography using 50 % acetonitrile in water (*v*/*v*) to obtain **5** as a colorless solid (16.9 mg, 0.0263 mmol, 99 %).

^{1}H-NMR (400 MHz, [D$_6$]DMSO): δ = 10.65 (s, 1H), 8.54 – 8.47 (m, 1H), 7.93 (dd, *J*=8.7, 3.1 Hz, 4H), 7.49 – 7.28 (m, 2H), 7.09 (tt, *J*=7.3 Hz, *J*=1.2 Hz, 1H), 7.03 – 6.88 (m, 4H), 6.69 (t, *J*=6.7 Hz, 1H), 6.61 (d, *J*=8.9 Hz, 2H), 6.46 (s, 2H), 4.19 (d, *J*=6.0 Hz, 2H), 3.78 ppm (d, *J*=6.2 Hz, 2H).

^{31}P-NMR (162 MHz, [D$_6$]DMSO): δ = -6.69 ppm.

^{13}C-NMR (101 MHz, [D$_6$]DMSO): δ = 170.0, 165.3, 157.6, 154.3, 151.1 (d, *J*=5.4 Hz), 140.2, 136.3, 129.9, 129.5, 122.7, 122.14, 122.05, 119.1, 117.7, 112.3, 111.2 ppm.

HRMS (ESI/TOF) m/z: [M - H]$^-$ calcd for $C_{28}H_{26}N_3O_{11}P_2^-$ 642.1048; found: 642.1067.

IR (KBr): $\tilde{\nu}$ = 3432, 3036, 1641, 1608, 1508, 1488, 1402, 1251, 1225, 1094, 1028, 925, 693, 556, 509 cm^{-1}.

UV/Vis (water): λ_{max} = 295, 213 nm.

R$_f$ (50 % acetonitrile/water, *v*/*v*) = 0.68.

MP: decomposition at 184 °C.

Tetrabenzyl (4-fluoro-1,3-phenylene) bis(phosphate) (**6a**)

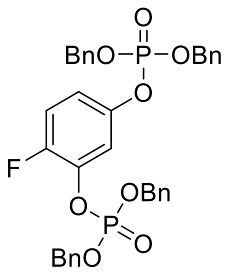

To a solution of 4-fluoro-1,3-dihydroxybenzene (70.0 mg, 0.546 mmol, 1.0 eq) in 1.5 mL anhydrous acetonitrile was added carbon tetrachloride (0.518 mL, 5.46 mmol, 10 eq), DIPEA (0.365 mL, 2.20 mmol, 4.0 eq.) and a catalytic amount of DMAP. The red solution was cooled to 0 °C and dibenzyl phosphite (0.276 mL, 1.62 mmol, 3.0 eq.) was added dropwise. The reaction mixture was stirred at 0 °C for 3 h before being quenched by the addition of 1.2 mL of a 0.5 M aqueous KH_2PO_4 solution. The organic and aqueous phases were separated and the aqueous phase repeatedly extracted with ethyl acetate. The combined organic layers were washed with brine, dried over Na_2SO_4, filtered and evaporated. The crude product was purified by two successive column chromatographies using 10 % methanol in DCM (*v*/*v*) and 30 % acetone in hexanes (*v*/*v*) as eluent to yield 147 mg (0.227 mmol, 43 %) **6a** as a colorless solid.

^{1}**H NMR** (400 MHz, $CDCl_3$) δ = 7.27-7.37 (m, 20H), 7.10-7.13 (m, 1H), 6.92-7.02 (m, 2H), 5.07-5.14 ppm (m, 8H).

^{31}**P NMR** (162 MHz, $CDCl_3$) δ = -6.3 (s, 1P), -6.5 ppm (s, 1P).

^{19}**F NMR** (377 MHz, $CDCl_3$) δ = -134.5 ppm.

^{13}**C NMR** (75 MHz, $CDCl_3$) δ = 150.8 (ddd, *J*=240.0, 5.6, 1.0 Hz), 146.6 – 145.4 (m), 135.2 (dd, *J*=6.5, 3.8 Hz), 129.2 – 127.8 (m), 117.5 – 117.3 (m), 117.0 (d, *J*=20.4 Hz), 115.1 – 114.8 (m), 70.3 (d, *J*=6.0 Hz), 70.2 ppm (d, *J*=5.8 Hz).

HRMS (ESI/TOF) m/z: $[M + Na]^+$ calcd for $C_{34}H_{31}FO_8P_2Na^+$ 671.1370; found: 671.1389.

IR (KBr): $\tilde{\nu}$ = 3645, 3442, 3089, 3066, 3034, 2959, 2894, 1507, 1286, 991 cm^{-1}.

UV/Vis (DCM): λ_{max} = 265, 226 nm.

$\mathbf{R_f}$ (30 % acetone/hexanes, *v*/*v*) = 0.23.

MP: 59 °C.

4-Fluoro-1,3-phenylene bis(dihydrogen phosphate) (**6**)

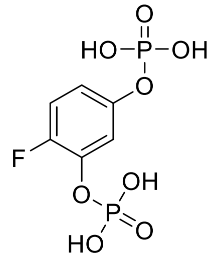

To a solution of **6a** (94.1 mg, 0.145 mmol, 1.0 eq.) in 25 mL ethanol was added a catalytic amount of Pd/C (10 wt-%, wet) under an Ar atmosphere. The Ar atmosphere was exchanged for H_2 and the reaction mixture stirred for 5 h 15 min at room temperature. The mixture was filtered through celite and the solvent was evaporated. The residue was taken up in a few mL of water and the solution was repeatedly washed with a total of 20 mL DCM. Evaporation of the aqueous phase afforded 18.9 mg (0.0656 mmol, 45 %) **6** as an off-white solid.

^{1}**H NMR** (400 MHz, D_2O) δ = 7.21 – 7.09 (m, 2H), 6.95 ppm (s, 1H).

^{19}**F NMR** (377 MHz, D_2O) δ = -136.83 ppm (bs).

^{31}**P NMR** (162 MHz, D_2O) δ = -4.14 ppm (bs).

^{13}**C NMR** (101 MHz, D_2O) δ = 150.9 (d, *J*=241.4 Hz), 147.3, 139.4, 117.0, 116.8, 115.1 ppm.

HRMS (ESI/TOF) m/z: $[M - H]^-$ calcd for $C_6H_6FO_8P_2^-$ 286.9527; found: 286.9525.

IR (KBr): $\tilde{\nu}$ = 3564, 3501, 2309, 1615, 1507, 1250, 1204, 989, 818, 689 cm^{-1}.

UV/Vis (D_2O): λ_{max} = 269 nm.

$\mathbf{R_f}$ (30 % acetonitrile/water, *v*/*v*) = 0.95.

MP: 122 - 125 °C.

2-Fluorobenzene-1,3-diol (**7a**)

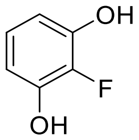

2-Fluoro-1,3-dimethoxybenzene (0.907 mL, 0.640 mmol, 1.0 eq) was dissolved in 1.5 mL anhydrous DCM. The solution was cooled to 0°C and a 1 M solution of BBr_3 in DCM (3.00 mL, 3.00 mmol, 4.7 eq) was added dropwise. The reaction mixture was stirred at room temperature for 5 h 15 min. 10 mL of water were added to quench the reaction and the mixture was extracted with ethyl acetate. The combined organic layers were dried over Na_2SO_4, filtered and evaporated. The crude product was purified by column chromatography using 5 % methanol in DCM (*v*/*v*) to yield 80.1 mg (0.624 mmol, 98 %) **7a** as an off-white solid.

^{1}**H NMR** (400 MHz, CD_3OD) 6.72 (td, *J* = 8.2, 1.8 Hz, 1H), 6.37 ppm (dd, *J* = 8.2, 7.6 Hz, 2H).

^{19}**F NMR** (377 MHz, CD_3OD) δ = -165.47 ppm (td, *J*=7.9, 1.8 Hz).

^{13}**C NMR** (101 MHz, CD_3OD) δ = 147.1 (d, *J*=10.7 Hz), 142.7 (d, *J*=234.8 Hz), 124.4 (d, *J*=4.5 Hz), 109.3 ppm.

HRMS (ESI/TOF) m/z: $[M - H]^-$ calcd for $C_6H_4FO_2^-$ 127.0201; found: 127.0200.

IR (KBr): $\tilde{\nu}$ = 3375, 3333, 2525, 2492, 2470, 2416, 1637, 1614, 1521, 1482, 1367, 1348, 1235, 1024 cm^{-1}.

UV/Vis (methanol): λ_{max} = 267, 203 nm.

$\mathbf{R_f}$ (5 % methanol/DCM, *v*/*v*) = 0.23.

MP: 111 °C (lit. 114-116 °C).[6]

Tetrabenzyl (2-fluoro-1,3-phenylene) bis(phosphate) (**7b**)

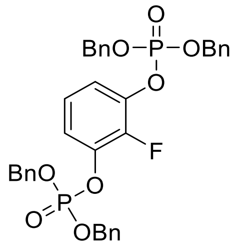

To a solution of **7a** (100 mg, 0.781 mmol, 1.0 eq) in 2 mL anhydrous acetonitrile was added carbon tetrachloride (0.748 mL, 7.81 mmol, 10 eq), DIPEA (0.539 mL, 3.12 mmol, 4.0 eq.) and a catalytic amount of DMAP. The solution was cooled to 0 °C and dibenzyl phosphite (0.398 mL, 2.34 mmol, 3.0 eq.) was added dropwise over 10 min. The reaction mixture was stirred at 0 °C for 3 h before being quenched by the addition of 1.6 mL of a 0.5 M aqueous KH_2PO_4 solution. The organic and aqueous phases were separated and the aqueous phase repeatedly extracted with ethyl acetate. The combined organic layers were washed with brine, dried over Na_2SO_4, filtered and evaporated. The crude product was purified by two successive column chromatographies using 50 % ethyl acetate in hexanes (*v*/*v)* and 1 % methanol in DCM (*v*/*v*) as eluent to yield 237 mg (0.366 mmol, 47 %) **7b** as a colorless solid.

^{1}H NMR (400 MHz, $CDCl_3$) δ = 7.38 – 7.29 (m, 20H), 7.10 (t, *J*=7.8 Hz, 2H), 6.95 – 6.88 (m, 1H), 5.14 ppm (d, *J*=8.4 Hz, 8H).

^{19}F NMR (377 MHz, $CDCl_3$) δ = -147.32 ppm (t, *J*=7.8 Hz).

^{31}P NMR (162 MHz, $CDCl_3$) δ = -6.5 ppm.

^{13}C NMR (101 MHz, $CDCl_3$) δ = 145.6 (dt, *J*=252.5, 5.9 Hz), 139.5 (dd, *J*=10.6, 7.1 Hz), 135.3 (d, *J*=7.0 Hz), 128.9, 128.8, 128.2, 123.6 (d, *J*=5.5 Hz), 118.6, 70.5 ppm (d, *J*=6.4 Hz).

HRMS (ESI/TOF) m/z: $[M + Na]^+$ calcd for $C_{34}H_{31}FO_8P_2Na^+$ 671.1370; found: 671.1403.

IR (KBr): $\tilde{\nu}$ = 3442, 3090, 3065, 3034, 2959, 2931, 2897, 1612, 1499, 1475, 1295, 1014 cm^{-1}.

UV/Vis (DCM): λ_{max} = 258, 207 nm.

R_f (1 % methanol/DCM, *v*/*v*) = 0.31.

MP: 45 °C.

2-Fluoro-1,3-phenylene bis(dihydrogen phosphate) (**7**)

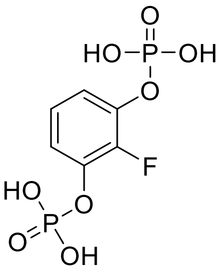

To a solution of **7b** (42.7 mg, 0.0658 mmol, 1.0 eq.) in 25 mL ethanol was added a catalytic amount of Pd/C (10 wt-%, wet) under an Ar atmosphere. The Ar atmosphere was exchanged for H_2 and the reaction mixture stirred for 2 h at room temperature. The mixture was filtered through celite and the solvent was evaporated. The residue was taken up in a few mL of water and the solution was repeatedly washed with a total of 10 mL DCM. Purification of the crude product by reversed-phase column chromatography using 10 % acetonitrile in water (*v*/*v*) yielded 18.5 mg (0.0642 mmol, 98 %) of **7** as a colorless solid.

^{1}H NMR (400 MHz, D_2O) δ = 7.12 ppm (br).

^{19}F NMR (282 MHz, D_2O) δ = -149.39 ppm.

^{31}P NMR (162 MHz, D_2O) δ = -3.81 ppm.

^{13}C NMR (101 MHz, D_2O) δ = 146.3 (d, *J*=246.9 Hz), 141.3 – 139.7 (m), 123.4 (d, *J*=2.1 Hz), 118.0 ppm.

HRMS (ESI/TOF) m/z: $[M - H]^-$ calcd for $C_6H_6FO_8P_2^-$ 286.9527; found: 286.9513.

IR (KBr): $\tilde{\nu}$ = 3629, 3616, 3441, 3235, 2921, 2853, 2380, 2347, 2314, 1615, 1213, 1164, 1061, 944, 809, 720, 674, 627, 539, 511, 502 cm^{-1}.

UV/Vis (DCM): λ_{max} = 235, 206 nm.

R_f (10 % acetonitrile/water, *v*/*v*) = 0.93.

MP: 125 °C.

1,3-Bis(bromomethyl)-2-fluorobenzene (**8a**)[7]

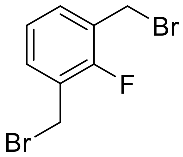

To a solution of 2-fluoro-1,3-dimethylbenzene (1.50 g, 12.1 mmol, 1.0 eq) in 24 mL $CHCl_3$ was added NBS (8.81 g, 49.5 mmol, 4.1 eq) and dibenzoyl peroxide (25 wt-% water, 311 mg, 1.03 mmol, 0.085 eq). The reaction mixture was stirred under reflux conditions over night followed by quenching with 70 mL of 3 % $Na_2S_2O_3$ solution. The phases were separated and the aqueous phase was extracted three times with DCM. The combined organic phases were dried over Na_2SO_4, filtered and the solvent was removed under reduced pressure. Purification of the crude product by column chromatography using 100 % hexanes afforded 668 mg (2.37 mmol, 20 %) **8a** as a colorless solid.

^{1}H NMR (400 MHz, $CDCl_3$) δ = 7.36 (t, *J*=7.5 Hz, 2H), 7.12 (t, *J*=8.0 Hz, 1H), 4.52 ppm (s, 4H), (lit. 7.36 (2H), 7.12 (1H), 4.52 ppm (4H)).

^{19}F NMR (377 MHz, $CDCl_3$) δ = -121.04 ppm.

^{13}C NMR (101 MHz, $CDCl_3$) δ = 158.6 (d, *J*=253.9 Hz; CF), 131.8 (d, *J*=3.4 Hz), 125.9 (d, *J*=14.6 Hz), 124.8 (d, *J*=4.6 Hz), 25.3 ppm (d, *J*=5.2 Hz), (lit. 159.8, 157.3, 131.73, 131.68, 125.8, 125.7, 124.7, 124.6, 25.23, 25.18 ppm).

HRMS (EI/70 eV) m/z: 199.9202 $[C_8H_6BrF^{\bullet}]^+$ (calculated 199.9631, 8 %), 200.9257 $[C_8H_7BrF]^+$ (calculated 200.9710, 8 %), 201.9124 $[C_8H_6BrF^{\bullet}]^+$ (calculated 201.9611, 8 %), 202.918 $[C_8H_7BrF]^+$ (calculated 202.9689, 7 %), 278.8152 $[M^{\bullet}]^+$ (calculated 278.8820, 11 %), 280.8200 $[M^{\bullet}]^+$ (calculated 280.8800, 22 %), 282.8094 $[M^{\bullet}]^+$ (calculated 282.8780, 11 %).

IR (KBr): $\tilde{\nu}$ = 3447, 3035, 1619, 1473, 1432, 1272, 1232, 1209, 1199, 1117, 1071, 874, 866, 822, 802, 742, 659, 551, 527 cm^{-1}.

UV/Vis (DCM): λ_{max} = 287 nm.

R$_f$ (hexanes) = 0.32.

MP: 91 °C, (lit. 90-91 °C).[7]

Tetraethyl ((2-fluoro-1,3-phenylene)bis(methylene))bis(phosphonate) (**8b**)

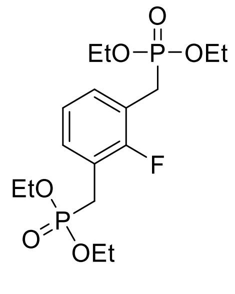

8a (304 mg, 1.08 mmol, 1.0 eq) was suspended in 2.77 mL $P(OEt)_3$ (16.2 mmol, 15 eq) and heated under reflux conditions until completion of the reaction as observed by TLC. After cooling to room temperature, the solution was evaporated to obtain the crude product as a yellow oil. Purification by column chromatography using 2 % methanol in DCM (*v*/*v*) afforded 385 mg (0.972 mmol, 90 %) **8b** as a colorless oil.

^{1}H NMR (400 MHz, [D$_6$]acetone) δ = 7.31 (tt, *J*=7.6 Hz, 2H), 7.09 (t, *J*=7.6 Hz, 1H), 4.07 – 3.95 (m, 8H), 3.19 (d, *J*=21.5 Hz, 4H; $ArCH_2$), 1.22 ppm (t, *J*=7.0 Hz, 12H; CH_2CH_3).

^{19}F NMR (377 MHz, [D$_6$]acetone) δ = -123.41 ppm.

^{31}P NMR (162 MHz, [D$_6$]acetone) δ = 23.45 ppm (d, *J*=4.3 Hz).

^{13}C NMR (101 MHz, [D$_6$]acetone) δ = 159.8 (d, *J*=250.6 Hz; CF), 131.4 (q, *J*=4.1 Hz), 124.4 (q, *J*=3.4 Hz), 121.1 – 120.5 (m), 62.7 – 62.2 (m), 26.6 (dd, *J*=139.1, 3.2 Hz), 16.9 – 16.6 ppm (m).

HRMS (ESI/TOF) m/z: $[M + H]^+$ calcd for $C_{38}H_{42}F_5N_3O_9P_2Na^+$ 864.2209; found: 864.2222.

IR (film): $\tilde{\nu}$ = 3465, 2984, 1472, 1254, 1052, 1025, 963, 546, 516, 486 cm^{-1}.

UV/Vis (methanol): λ_{max} = 305, 272, 267, 225 nm.

R$_f$ (2 % methanol/DCM, *v*/*v*) = 0.16.

((2-Fluoro-1,3-phenylene)bis(methylene))bis(phosphonic acid) (**8**)

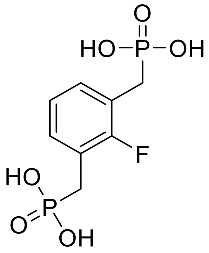

Phosphonate **8b** (92.5 mg, 0.292 mmol, 1.0 eq) was dissolved in 4.5 mL anhydrous DCM and kept at 0 °C for 30 min before the dropwise addition of 0.535 mL TMSBr (4.09 mmol, 14 eq). The reaction mixture was allowed to warm to room temperature and stirred for two days. After completion of the deprotection as indicated by TLC, the reaction was quenched with 5 mL methanol and evaporated *in vacuo*. The remaining residue was evaporated two times from 5 mL methanol and three times 5 mL DCM to obtain 62.7 mg (0.221 mmol, 75 %) **8** as a colorless solid.

^{1}H NMR (400 MHz, [D$_6$]DMSO) δ = 7.21 (t, *J*=7.5 Hz, 2H), 7.03 (t, *J*=7.5 Hz, 1H), 2.96 ppm (d, *J*=21.5 Hz, 4H).

^{19}F NMR (377 MHz, [D$_6$]DMSO) δ = -122.42 ppm.

^{31}P NMR (162 MHz, [D$_6$]DMSO) δ = 19.35 ppm.

^{13}C NMR (101 MHz, D_2O) δ = 158.6 (d, *J*=244.5 Hz; CF), 130.2 – 129.6 (m), 124.0 – 123.7 (m), 121.6 – 120.8 (m), 28.3 ppm (dd, *J*=130.7, 1.7 Hz; *C*F$_2$).

HRMS (ESI/TOF) m/z: [M - H]$^-$ calcd for $C_8H_{10}FO_6P_2^-$ 282.9942; found: 282.9920.

IR (KBr): $\tilde{\nu}$ = 3454, 2988, 2948, 2815, 2234, 2148, 1635, 1473, 1412, 1193, 1178, 1136, 1102, 1024, 967, 957, 727, 513, 477, 448 cm^{-1}.

UV/Vis (H_2O): λ_{max} = 272, 266, 227 nm.

R$_f$ (5 % methanol/DCM, *v*/*v*) = 0.28.

MP: decomposition at 129 °C.

Tetraethyl ((2-fluoro-1,3-phenylene)bis(difluoromethylene))bis(phosphonate) (**9a**)

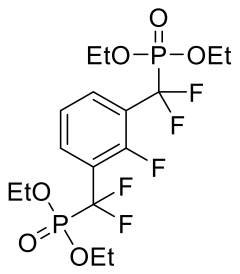

At -78 °C, 7.98 mL (7.98 mmol, 5.5 eq) of a 1 M NaHMDS solution in THF were diluted in 50 mL anhydrous THF. The resulting solution was kept at -78 °C for 30 min before the dropwise addition over 5 min of 575 mg (1.45 mmol, 1.0 eq) **8b** dissolved in 15 mL anhydrous THF. After 1 h at -78 °C, 3.34 g (10.6 mmol, 7.3 eq) NFSI dissolved in 10 mL anhydrous THF were added dropwise over 5 min. The resulting green solution was kept at -78 °C for 2 h, followed by the addition of 50 mL of an aqueous 0.01 M HCl solution at room temperature to quench the reaction. Volatiles were removed *in vacuo* and the remaining aqueous suspension was extracted four times with DCM. The combined organic extracts were washed with saturated bicarbonate solution, dried over Na_2SO_4, filtered and evaporated. Purification of the crude product by column chromatography using a gradient of 15 % to 25 % acetone in hexanes (*v*/*v*) afforded 360 mg (0.769 mmol, 53 %) **9a** as a colorless oil.

^{1}H NMR (400 MHz, $CDCl_3$) δ = 7.70 (t, *J*=7.3 Hz, 2H), 7.33 (t, *J*=7.5 Hz, 1H), 4.36 – 4.18 (m, 8H;), 1.35 ppm (t, *J*=6.9 Hz, 12H; CH$_2$C*H*$_3$).

^{19}F NMR (377 MHz, $CDCl_3$) δ = -107.06 (dd, *J*=110.5, 22.0 Hz; 4F; CF$_2$), -112.03 ppm (d, *J*=22.0 Hz, 1F; CF).

^{31}P NMR (162 MHz, $CDCl_3$) δ = 4.91 ppm (d, *J*=110.5 Hz).

^{13}C NMR (75 MHz, $CDCl_3$) δ = 157.5 (d, *J*=269.2 Hz; CF), 132.4 – 131.2 (m), 124.1, 122.7 – 121.2 (m), 116.4 (td, *J*=264.3, 219.2 Hz), 65.4 (d, *J*=7.2 Hz), 16.4 ppm (d, *J*=5.9 Hz).

HRMS (ESI/TOF) m/z: $[M + Na]^+$ calcd for $C_{16}H_{23}F_5O_6P_2Na^+$ 491.0782; found: 491.0794.

IR (film): $\tilde{\nu}$ = 3562, 3508, 3219, 3097, 2987, 2936, 2915, 2873, 2838, 1619, 1590, 1467, 1447, 1394, 1372, 1275, 1241, 1164, 1137, 1051, 1021, 973, 941, 806, 750, 583, 562, 531 cm^{-1}.

UV/Vis (DCM): λ_{max} = 227, 268, 274 nm.

R_f (40 % acetone/hexanes, *v*/*v*) = 0.49.

<u>((2-Fluoro-1,3-phenylene)bis(difluoromethylene))bis(phosphonic acid) (**9**)</u>

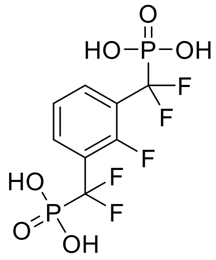

Phosphonate **9a** (266 mg, 0.567 mmol, 1.0 eq) was dissolved in 8.3 mL anhydrous DCM and kept at 0 °C for 30 min before the dropwise addition of 1.04 mL TMSBr (7.9 mmol, 14 eq). The reaction mixture was allowed to warm to room temperature and stirred for two days. After completion of the deprotection as indicated by TLC, the reaction was quenched with 5 mL methanol and evaporated *in vacuo*. The remaining residue was evaporated two times from 5 mL methanol and three times 5 mL DCM to obtain 204 mg (0.573 mmol, quant.) **9** as a colorless solid.

^{1}H NMR (400 MHz, CD_3OD) δ = 7.7 (t, *J*= 7.6 Hz, 2H), 7.4 ppm (t, *J*=7.6 Hz, 1H).

^{19}F NMR (377 MHz, CD_3OD) δ = -110.00 (dd, *J*=107.3, 22.6 Hz, 4F; CF_2), -113.22 ppm (t, *J*=22.6 Hz, 1F; CF).

^{31}P NMR (162 MHz, CD_3OD) δ = 3.04 ppm.

^{13}C NMR (101 MHz, CD_3OD) δ = 158.8 (d, *J*=266.2 Hz; CF), 132.9 – 132.1 (m), 124.8, 124.4 – 123.5 (m), 118.9 ppm (td, *J*=263.0, 211.7 Hz).

HRMS (ESI/TOF) m/z: $[M - H]^-$ calcd for $C_8H_6F_5O_6P_2^-$ 354.9565; found: 354.9566.

IR (KBr): $\tilde{\nu}$ = 3605, 3591, 3583, 3572, 3452, 3444, 1730, 1713, 1695, 1681, 1673, 1667, 1660, 1650, 1623, 1465, 1052, 983, 971, 924, 738, 564, 555 cm^{-1}.

UV/Vis (methanol): λ_{max} = 273, 267, 217 nm.

R$_f$ (40 % water/acetonitrile, *v*/*v*) = 0.89.

MP: 137 °C.

3,5-Bis(bromomethyl)-4-fluorobenzonitrile (**10b**)

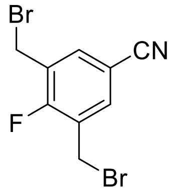

To a solution of 4-fluoro-3,5-dimethylbenzonitrile (**10a**, 1.4 g, 9.38 mmol, 1.0 eq) in 28 mL chloroform were added NBS (6.85 g, 38.5 mmol, 4.1 eq) and benzoyl peroxide (25 wt-% water, 242 mg, 0.798 mmol, 0.085 eq). The resulting suspension was stirred overnight under reflux conditions followed by reduction *in vacuo*. The remaining solution was repeatedly washed with a total of 150 mL of an aqueous 3 % $Na_2S_2O_3$ solution and the combined aqueous phases were extracted with DCM. The combined organic phases were dried over Na_2SO_4, filtered and evaporated to obtain a viscous oil. Purification of the crude product *via* column-chromatography using a gradient of 1 % acetone to 2 % acetone in hexanes (*v*/*v*) afforded 717 mg (2.37 mmol, 25 %) **10b** as a colorless solid.

^{1}H NMR (400 MHz, [D$_6$]acetone) δ = 8.02 (d, *J*=6.5 Hz, 2H), 4.72 ppm (d, *J*=1.1 Hz, 4H; $(CH_2)_2$).

^{19}F NMR (377 MHz, [D$_6$]acetone) δ = -113.70 ppm (t, *J*=6.5 Hz).

^{13}C NMR APT (101 MHz, [D$_6$]acetone) δ = 162.1 (d, *J*=262.2 Hz; CF), 136.6 (d, *J*=5.6 Hz), 129.2 (d, *J*=16.0 Hz), 117.7, 109.9 (d, *J*=4.4 Hz), 24.6 ppm (d, *J*=4.7 Hz).

HRMS (EI, 70 eV) m/z (%): 74 $[C_9H_6FN]^{2+}$ (calculated 73.5237, 27 %), 226 $[C_9H_6BrFN]^+$ (calculated 225.9662, 100 %), 228 $[C_9H_6BrFN]^+$ (calculated 227.9642, 98 %), 304.8845 $[M^{\bullet}]^+$ (calculated 304.8846, 5 %), 306.8817 $[M^{\bullet}]^+$ (calculated 306.8825, 10 %), 308.8800 $[M^{\bullet}]^+$ (calculated 308.8810, 5 %).

IR (KBr): $\tilde{\nu}$ = 3435, 3068, 3039, 2990, 2924, 2857, 2229, 1801, 1724, 1668, 1634, 1615, 1482, 1246, 898 cm^{-1}.

UV/Vis (DCM): λ_{max} = 288, 232 nm.

R_f (10 % acetone/hexanes, *v*/*v*) = 0.26.

MP: 109 °C.

Tetraethyl ((5-cyano-2-fluoro-1,3-phenylene)bis(methylene))bis(phosphonate) (**10c**)

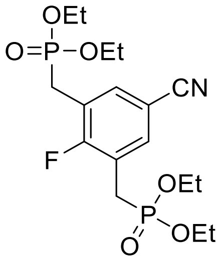

10b (309 mg, 1.01 mmol, 1.0 eq) was suspended in 2.6 mL $P(OEt)_3$ (15.2 mmol, 15 eq) and heated under reflux conditions until completion of the reaction as observed by TLC. After cooling to room temperature, the solution was evaporated to obtain the crude product as a yellow oil. Purification by column chromatography using a gradient of 1 % to 5 % methanol in DCM (*v*/*v*) afforded 389 mg (0.923 mmol, 92 %) **10c** as a colorless solid.

^{1}H NMR (400 MHz, $CDCl_3$) δ = 7.59 – 7.55 (m, 2H), 4.06 (m, 4H), 3.18 (d, *J*=21.8 Hz, 4H; CH_2), 1.26 ppm (t, *J*=7.3 Hz, 12H; CH_2CH_3).

^{19}F NMR (377 MHz, $CDCl_3$) δ = -111.30 ppm (t, *J*=5.1 Hz).

^{31}P NMR (162 MHz, $CDCl_3$) δ = 23.25 ppm (d, *J*=5.1 Hz).

^{13}C NMR APT (101 MHz, $CDCl_3$) δ = 161.5 (dt, *J*=249.4, 6.9 Hz; CF), 134.7 – 134.5 (m), 122.3 – 121.5 (m), 117.9, 108.7 (d, *J*=4.0 Hz), 62.7 – 62.4 (m), 26.6 (dd, J=143.5, 3.1 Hz; PCH_2), 16.6 – 16.1 ppm (m).

HRMS (ESI/TOF) m/z: $[M + Na]^+$ calcd for $C_{17}H_{26}FNO_6P_2Na^+$ 444.1112; found: 444.1129.

IR (KBr): $\tilde{\nu}$ = 3551, 3457, 3088, 2982, 2931, 2915, 2872, 2229, 1635, 1606, 1511, 1479, 1247, 1056, 1023 cm^{-1}.

UV/Vis (DCM): λ_{max} = 273, 234 nm.

R_f (5 % methanol/DCM, *v*/*v*) = 0.36.

MP: 78 °C.

Tetraethyl ((5-cyano-2-fluoro-1,3-phenylene)bis(difluoromethylene)) bis(phosphonate) (**10d**)

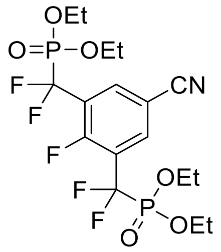

At -78 °C, 6.53 mL (6.53 mmol, 5.5 eq) of a 1 M NaHMDS solution in THF were diluted in 41 mL anhydrous THF. The resulting solution was kept at -78 °C for 30 mins before the dropwise addition over 10 min of 500 mg (1.19 mmol, 1.0 eq) **10c** dissolved in 12 mL anhydrous THF. After 5 min at -78 °C, 2.73 g (8.66 mmol, 7.3 eq) NFSI dissolved in 12 mL anhydrous THF was added dropwise over 10 min. The resulting brown solution was kept at -78 °C for 2 h, followed by the addition of 41 mL of an aqueous 0.01 M HCl solution at room temperature to quench the reaction. Volatiles were removed *in vacuo* and the remaining aqueous suspension was extracted four times with DCM. The combined organic extracts were washed with saturated bicarbonate solution, dried over Na_2SO_4, filtered and evaporated. Purification of the crude product by column chromatography using 25 % acetone in hexanes (*v*/*v)* afforded 206 mg (0.417 mmol, 35 %) **10d** as a colorless solid.

^{1}H NMR (400 MHz, $CDCl_3$) δ = 7.97 (d, *J*=5.8 Hz, 2H; Ar-H), 4.38 – 4.20 (m, 8H; CH_2CH_3), 1.36 ppm (t, *J*=7.0 Hz, 3H; CH_2CH_3).

^{19}F NMR (377 MHz, $CDCl_3$) δ = -101.64 (t, *J*=19.9 Hz, 1F; CF), -107.97 ppm (dd, *J*=107.9, 19.9 Hz, 4F; CF_2).

^{31}P NMR (162 MHz, $CDCl_3$) δ = 3.82 ppm (t, *J*=107.9 Hz).

^{13}C NMR ^{1}H decoupled (101 MHz, $CDCl_3$) δ = 159.7 (d, *J*=275.8 Hz; CF), 135.6 (Ar-CH), 124.2 (hept, *J*=10.1 Hz; *C*CF_2), 116.3 (*C*CN), 116.2 (td, *J*=264.4, 219.9 Hz; C*C*F_2), 109.4 (CN), 65.7 (d, *J*=6.9 Hz; *C*H_2CH_3), 16.4 ppm (d, J=5.5 Hz; CH_2*C*H_3).

^{13}C NMR ^{19}F decoupled (101 MHz, $CDCl_3$) δ = 159.7 (CF), 135.5 (ddt, *J*=179.0, 7.1, 1.7 Hz; Ar-H), 124.3 (dd, *J*=14.2, 1.3 Hz; *C*CF_2), 116.3 (t, *J*=6.0 Hz; *C*CN), 116.2 (d, *J*=219.9 Hz; C*C*F_2), 109.4 (CN), 65.7 (t, *J*=149.8 Hz; *C*H_2CH_3), 16.4 ppm (q, J=127.7 Hz; CH_2*C*H_3).

HRMS (ESI/TOF) m/z: $[M + Na]^+$ calcd for $C_{17}H_{22}F_5NO_6P_2Na^+$ 516.0735; found: 516.0740.

IR (KBr): $\tilde{\nu}$ = 3449, 3217, 3062, 2989, 2922, 2854, 2239, 1896, 1608, 1477, 1272, 1020 cm^{-1}.

UV/Vis (chloroform): λ_{max} = 283, 275, 239 nm.

R_f (40 % acetone/hexanes, *v*/*v*) = 0.31.

MP: 52 °C.

Tert-butyl (3,5-bis((diethoxyphosphoryl)difluoromethyl)-4-fluorobenzyl)carbamate (**10e**)

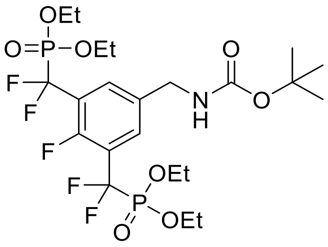

Nitrile **10d** (300 mg, 0.608 mmol, 1.0 eq) was dissolved in 4.5 mL dry methanol and the resulting solution cooled to 0 °C. After having kept the reaction mixture at 0 °C for 10 min, 266 mg (1.22 mmol, 2.0 eq) di-*tert*-butyl dicarbonate and 14.5 mg (0.0681 mmol, 0.1 eq) $NiCl_2$ x 6 H_2O were added, followed by 161 mg (4.26 mmol, 7.0 eq) $NaBH_4$ over 30 min.[8] The black mixture was allowed to warm to room temperature and stirred overnight followed by the addition of 0.129 mL diethylenetriamine. After stirring for 30 min, volatiles were removed under reduced pressure and the residue was taken up in ethyl acetate. The solution was washed two times with saturated bicarbonate solution, dried over Na_2SO_4, filtered and evaporated to obtain 309 mg (0.517 mmol, 85 %) **10e** as a colorless oil. The crude product was used in subsequent reactions without further purification.

^{1}H NMR (400 MHz, [D$_6$]acetone) δ = 7.71 (d, *J*=5.0 Hz, 2H), 4.37 (d, *J*=6.5 Hz, 2H), 4.30 – 4.16 (m, 8H), 1.42 (s, 9H), 1.31 ppm (t, *J*=7.1 Hz, 12H).

^{19}F NMR (377 MHz, [D$_6$]acetone) δ = -108.56 (dd, *J*=109.6, 19.5 Hz, 4F; CF_2), -116.63 ppm (t, *J*=19.5 Hz, 1 F; CF).

^{31}P NMR (162 MHz, [D$_6$]acetone) δ = 3.48 ppm (t, *J*=109.6 Hz).

^{13}C NMR (101 MHz, [D$_6$]acetone) δ = 156.000, 155.998 (d, *J*=261.3 Hz, CF), 137.1, 130.2, 122.9 – 120.8 (m), 116.5 (td, *J*=262.3, 216.9 Hz), 78.4, 64.7 (d, *J*=6.6 Hz), 42.8, 27.7, 15.8 ppm (d, *J*=5.2 Hz).

HRMS (ESI/TOF) m/z: $[M + Na]^+$ calcd for $C_{22}H_{34}F_5NO_8P_2Na^+$ 620.1572; found: 620.1568.

IR (film in $CHCl_3$): $\tilde{\nu}$ = 3333, 2983, 2934, 1711, 1518, 1479, 1446, 1368, 1274, 1166, 1096, 1022, 779, 543 cm^{-1}.

UV/Vis (methanol): λ_{max} = 280, 273, 206 nm.

R_f (40 % acetone/hexanes, *v*/*v*) = 0.32.

(3,5-Bis((diethoxyphosphoryl)difluoromethyl)-4-fluorophenyl)methanaminium chloride (**10e’**)

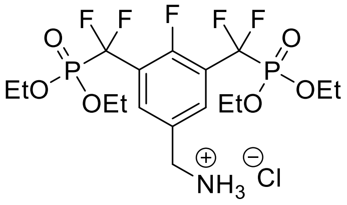

Boc-protected amine **10e** (312 mg, 0.522 mmol, 1.0 eq) was kept at 0 °C under an Ar atmosphere in a round-bottom flask for 15 min before the addition of 13.1 mL of a 4 M HCl solution in dioxane (52.2 mmol, 100 eq). The solution was allowed to warm to room temperature and stirred until complete deprotection was observed by TLC (25-35 min). The reaction mixture was evaporated without contact to air and the colorless solid obtained was used in subsequent reactions without further purification.

^{1}H NMR (400 MHz, CD_3OD) δ = 7.91 (d, *J*=5.9 Hz, 2H), 4.44 – 4.26 (m, 8H), 4.26 (s, 2H), 1.37 ppm (t, *J*=7.1 Hz, 12H).

^{19}F NMR (377 MHz, CD_3OD) δ = -109.53 (dd, *J*=110.7, 21.2 Hz, 4F; CF_2), -112.82 ppm (t, *J*=21.2 Hz, 1 F; CF).

^{31}P NMR (162 MHz, CD_3OD) δ = 3.65 ppm (t, *J*=110.7 Hz).

HRMS (ESI/TOF) m/z: $[M + H]^+$ calcd for $C_{17}H_{27}F_5NO_6P_2^+$ 498.1228; found: 498.1239.

R_f (10 % methanol/DCM, *v*/*v*) = 0.21.

Tetraethyl ((2-fluoro-5-((2-((4-((4-phenoxyphenyl)carbamoyl)phenyl)amino) acetamido)methyl)-1,3-phenylene) bis(difluoromethylene))bis(phosphonate) (**10f**)

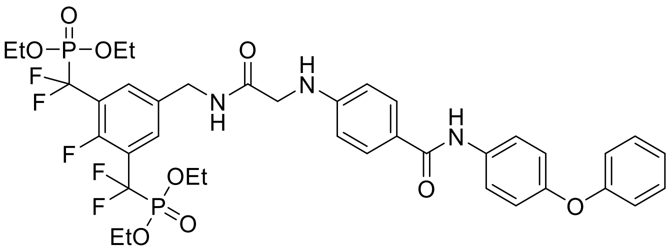

5e (189 mg, 0.522 mmol, 1.0 eq) was suspended in 1 mL anhydrous DCM and kept at 0 °C for 10 min. After the addition of EDC-HCl (100 mg, 0.522 mmol, 1.0 eq) and HOBt (70.0 mg, 0.522 mmol, 1.0 eq), the suspension was stirred for 3 min followed by the addition of NEt_3 (0.451 mL, 3.23 mmol, 6.2 eq) and **10e’** (279 mg, 0.522 mmol, 1.0 eq) suspended in 1 mL anhydrous DCM. The reaction mixture was allowed to warm to room temperature and stirred overnight. The mixture was quenched with 4 mL of a saturated NH_4Cl solution and extracted

eight times with ethyl acetate. The combined organic extracts were dried over Na_2SO_4, filtered and evaporated. Purification of the crude product by column chromatography using a gradient of 1 % to 10 % methanol in DCM (*v*/*v*) afforded 193 mg **10f** (0.230 mmol, 44 % over 2 steps) as a colorless solid.

^{1}H NMR (400 MHz, $CDCl_3$) δ = 8.68 (bs, 1H), 7.80 (d, *J*=8.7 Hz, 2H), 7.67 (d, *J*=9.2 Hz, 2H), 7.53 (d, *J*=6.5 Hz, 2H), 7.32 (t, *J*=7.5 Hz, 2H), 7.15 – 7.04 (m, 2H), 7.03 – 6.96 (m, 4H), 6.59 (d, *J*=8.7 Hz, 2H), 4.74 (bs, 1H), 4.51 (d, *J*=6.3 Hz, 2H), 4.31 – 4.16 (m, 8H), 3.91 (d, *J*=5.9 Hz, 2H; CH_2), 1.32 ppm (t, *J*=7.3 Hz, 12H; CH_2CH_3).

^{19}F NMR (377 MHz, $CDCl_3$) δ = -106.62 (dd, *J*=111.4, 20.2 Hz, 4F; CF_2), -113.90 ppm (t, *J*=20.2 Hz; 1F; CF).

^{31}P NMR (162 MHz, $CDCl_3$) δ = 4.40 ppm (t, *J*=111.4 Hz).

^{13}C NMR (76 MHz, [D$_6$]acetone) δ = 165.9, 159.0, 157.2, 155.0, 153.2, 151.9, 136.9, 131.9 – 131.0 (m), 130.7, 130.0, 124.6, 123.7, 122.4, 120.2, 118.8, 112.6, 65.9 (d, *J*=6.9 Hz), 48.1, 42.4, 16.7 ppm (d, *J*=5.2 Hz).

HRMS (ESI/TOF) m/z: $[M + Na]^+$ calcd for $C_{38}H_{42}F_5N_3O_9P_2Na^+$ 864.2209; found: 864.2222.

IR (KBr): $\tilde{\nu}$ = 3435, 1645, 1609, 1508, 1488, 1264, 1225, 1024, 872, 751 cm^{-1}.

UV/Vis (DCM): λ_{max} = 317 nm.

R_f (5 % methanol/DCM, *v*/*v*) = 0.29.

MP: decomposition at 198 °C.

((2-Fluoro-5-((2-((4-((4-phenoxyphenyl)carbamoyl)phenyl)amino)acetamido) methyl)-1,3-phenylene)bis(difluoromethylene))bis(phosphonic acid) (**10**)

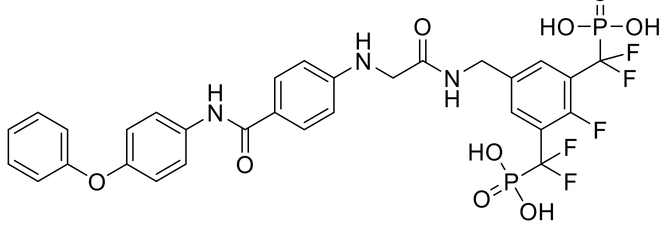

10f (76.0 mg, 0.090 mmol, 1.0 eq) was dissolved in 2.6 mL anhydrous DCM and kept at 0 °C for 10 min before the dropwise addition of 0.165 mL TMSBr (1.26 mmol, 14 eq). The reaction mixture was allowed to warm to room temperature and stirred for two days. After completion of the deprotection as indicated by TLC, the reaction was quenched with 5 mL methanol and

evaporated *in vacuo*. The remaining residue was evaporated two times from 5 mL methanol and three times 5 mL DCM. Purification of the crude product by reversed-phase column chromatography using 15 % acetonitrile in water (*v*/*v*) afforded 49.6 mg (0.0680 mmol, 76 %) **10** as a colorless solid.

^{1}H NMR (400 MHz, CD_3OD) δ = 7.83 (d, *J*=8.4 Hz, 2H; Ar-H), 7.66 (d, *J*=8.4 Hz, 2H; Ar-H), 7.54 (d, *J*=5.4 Hz, 2H; Ar-H), 7.34 (t, *J*=7.3 Hz, 2H; Ar-H), 7.08 (t, *J*=7.2 Hz, 1H; Ar-H), 7.02 – 6.93 (m, 4H; Ar-H), 6.70 (d, *J*=8.4 Hz, 2H; Ar-H), 4.43 (s, 2H; CH_2), 3.92 ppm (s, 2H; CH_2).

^{19}F NMR (377 MHz, CD_3OD) δ = -107.53 (dd, *J*=92.7, 12.5 Hz; 4F; CF_2), -115.6 – -116.2 ppm (m, 1F; CF).

^{31}P NMR (162 MHz, CD_3OD) δ = 2.94 ppm (t, *J*=92.7 Hz).

^{13}C NMR (101 MHz, CD_3OD) δ = 170.6, 167.1, 157.7, 153.7, 148.4, 134.6 (d, *J*=3.2 Hz), 134.1, 130.2 – 129.9 (m), 129.4, 129.2, 125.6, 122.79, 122.77, 118.8, 118.1, 114.0, 53.4, 41.6 ppm.

HRMS (ESI/TOF) m/z: $[M - H]^-$ calcd for $C_{30}H_{25}F_5N_3O_9P_2^-$ 728.0992; found: 728.1008.

IR (KBr): $\tilde{\nu}$ = 3435, 1645, 1609, 1508, 1488, 1316, 1264, 1225, 1148, 1070, 1024, 751 cm^{-1}.

UV/Vis (methanol): λ_{max} = 307 nm.

R_f (40 % acetonitrile/water, *v*/*v*) = 0.58.

MP: 159 °C.

Molecular docking

Compounds were docked into the previously published homology model of STAT5b[9] using AutoDockFR.[10] STAT5b amino acids Lys600, Arg618, Ser620, Asp621, Ser622, Glu623 and Met644 were defined as flexible.

NMR spectra

^{1}H NMR of compound **4**

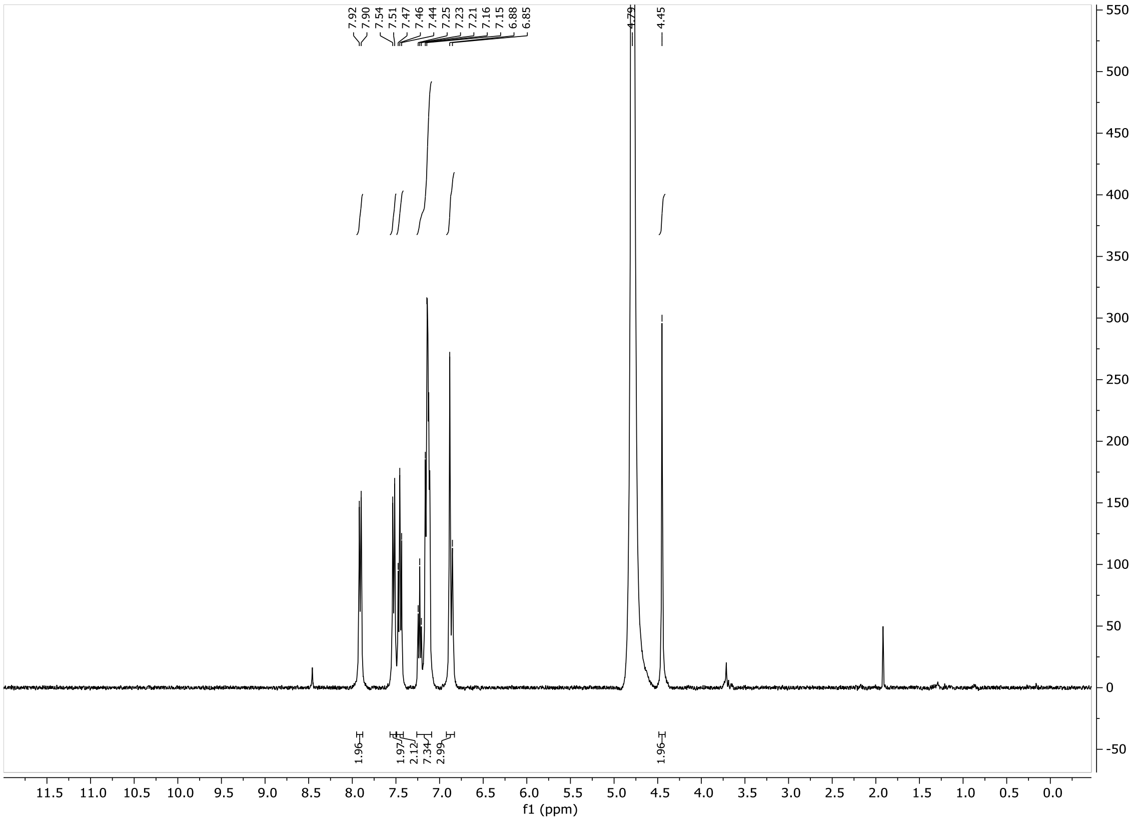

^{31}P NMR of compound **4**

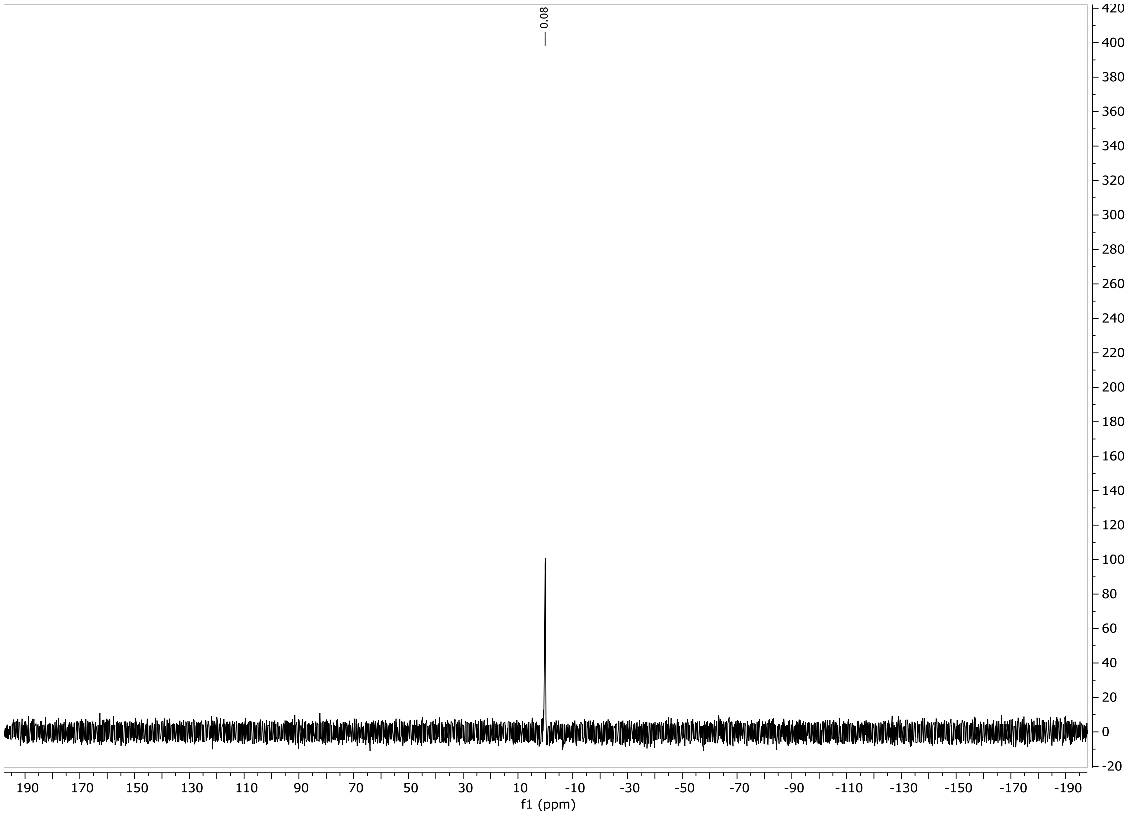

^{13}C NMR of compound **4**

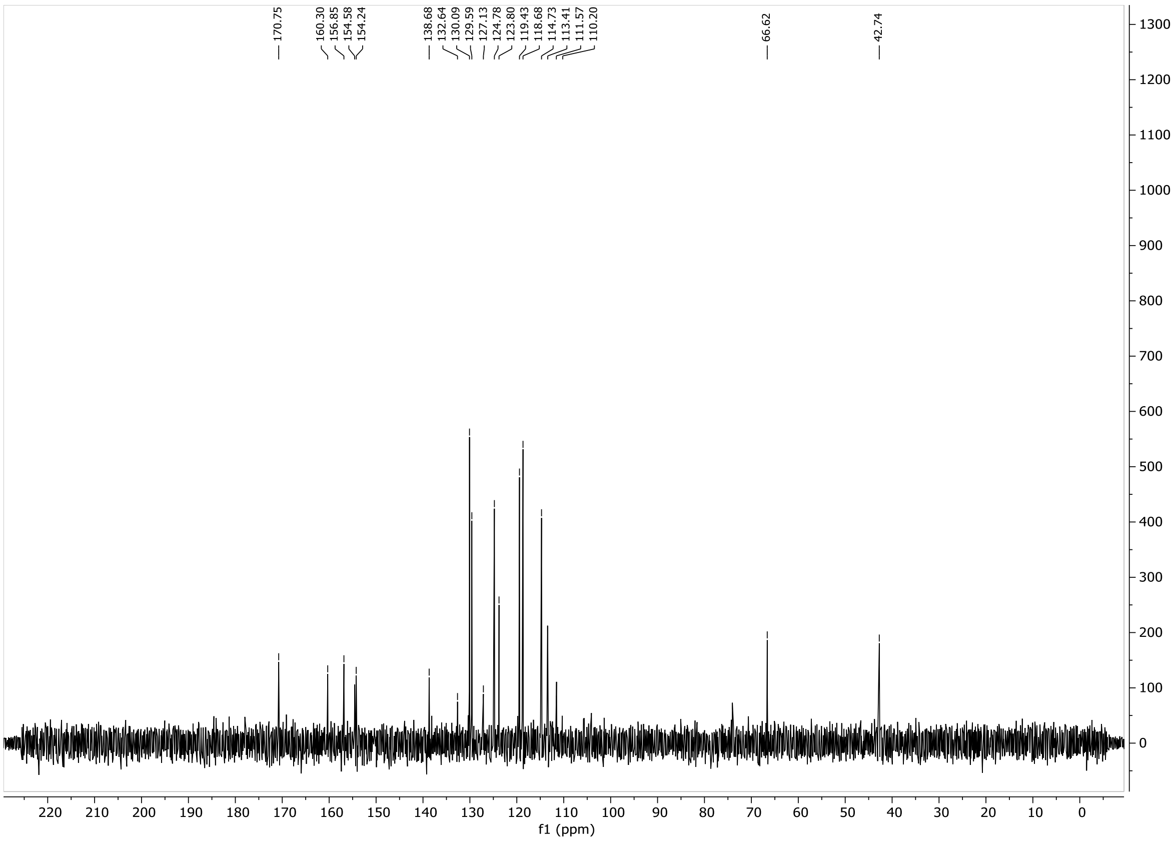

^{1}H NMR of compound **5**

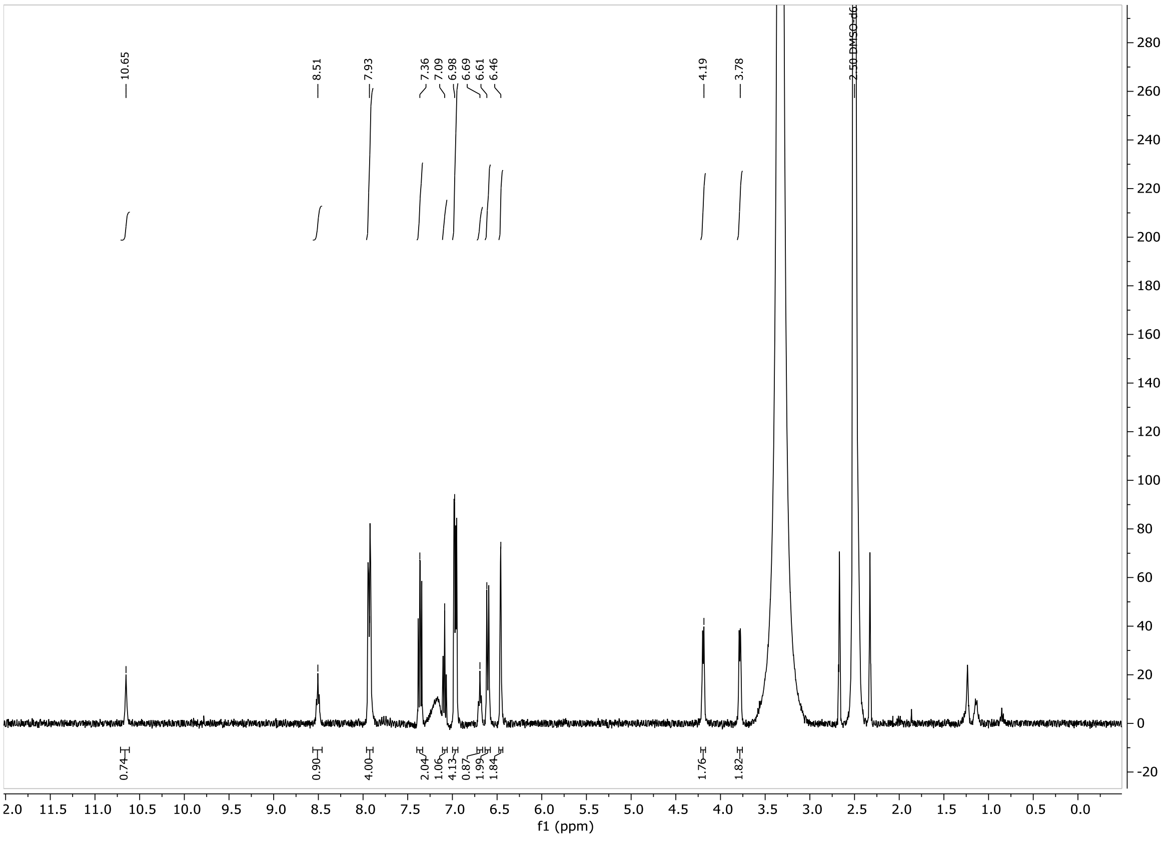

^{31}P NMR of compound **5**

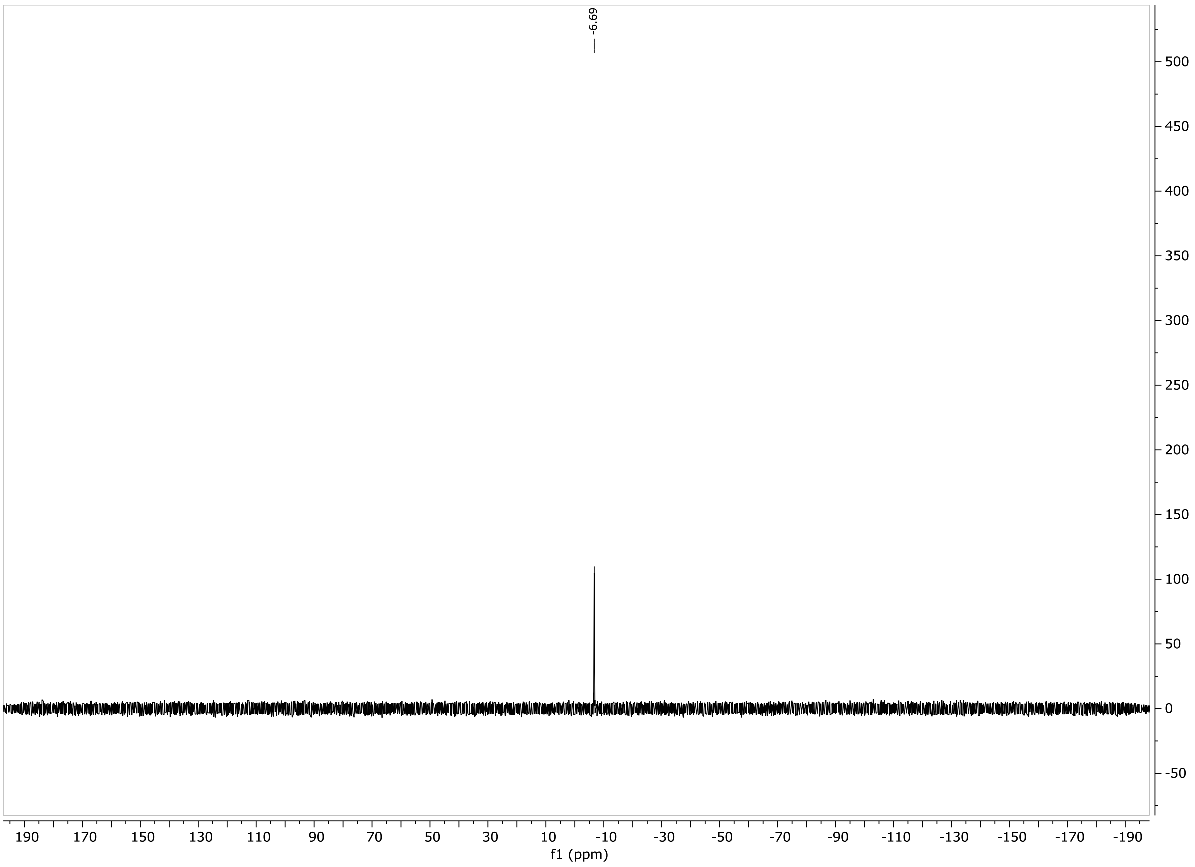

^{13}C NMR of compound **5**

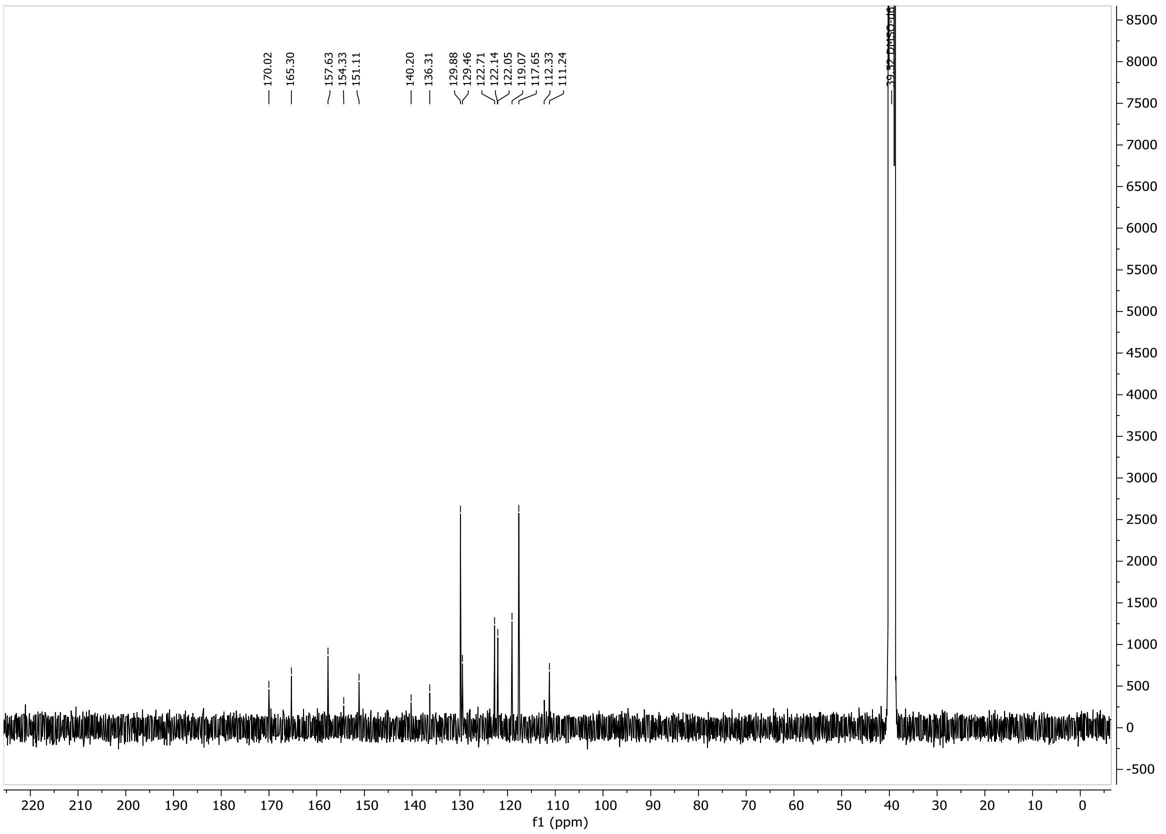

^{1}H NMR of compound **10**

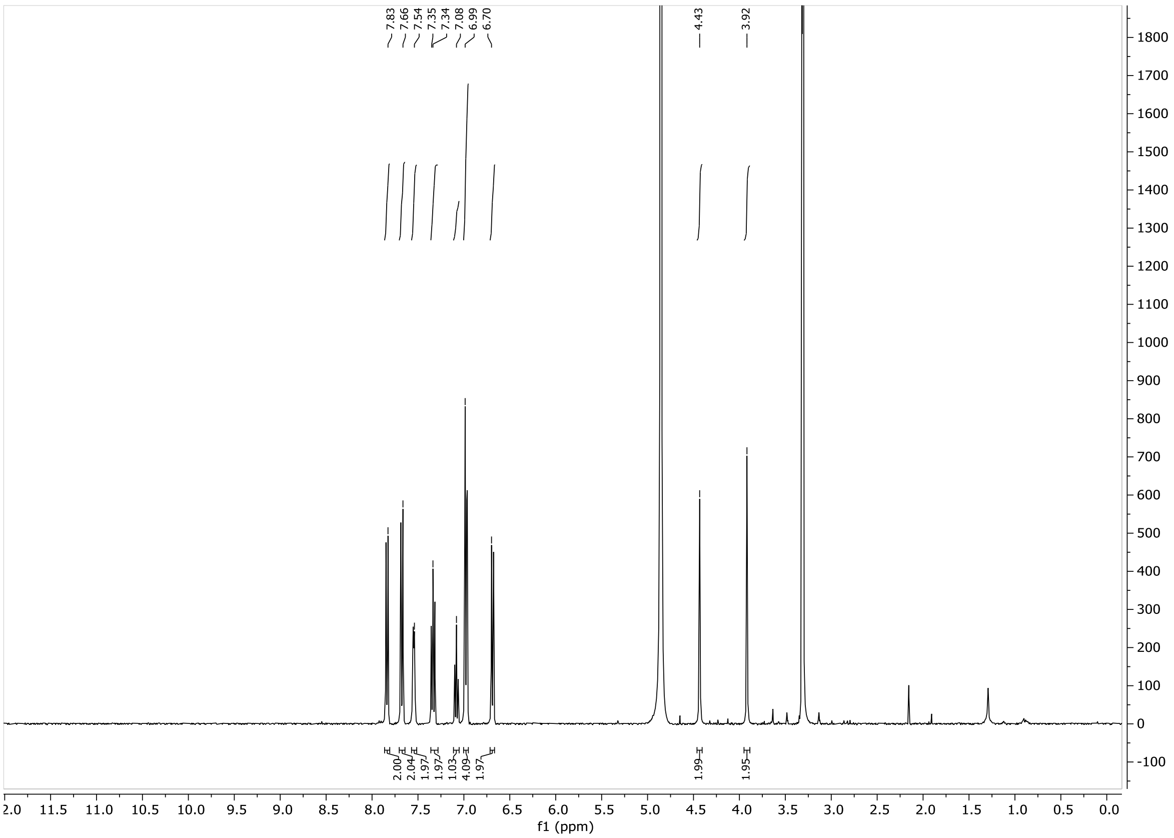

^{19}F NMR of compound **10**

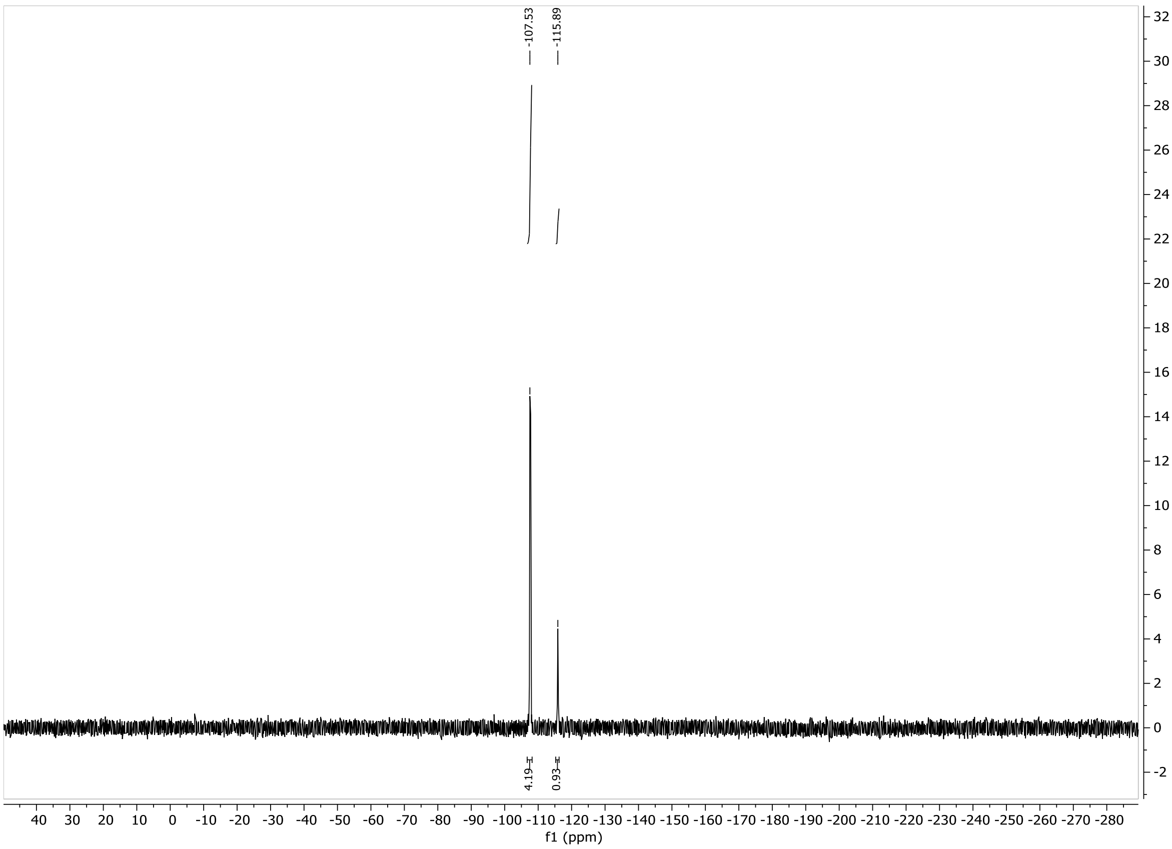

^{31}P NMR of compound **10**

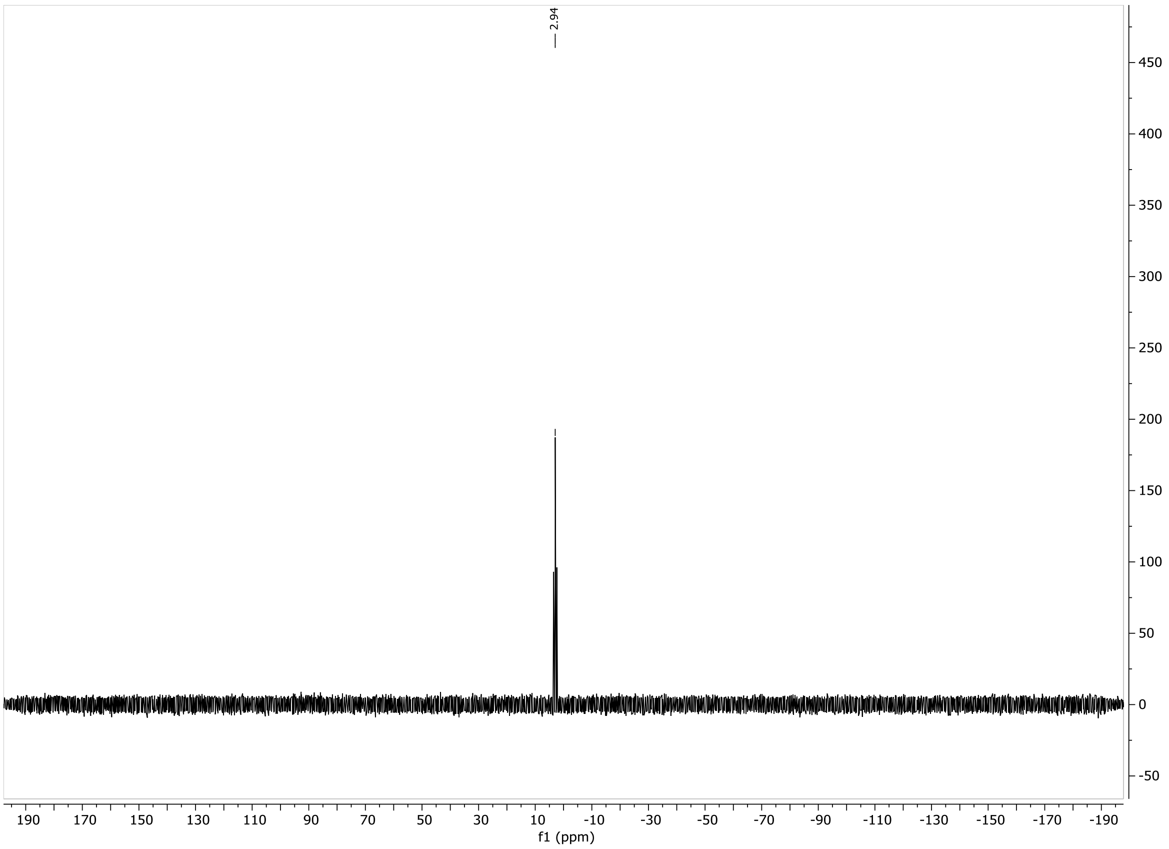

^{13}C NMR of compound **10**

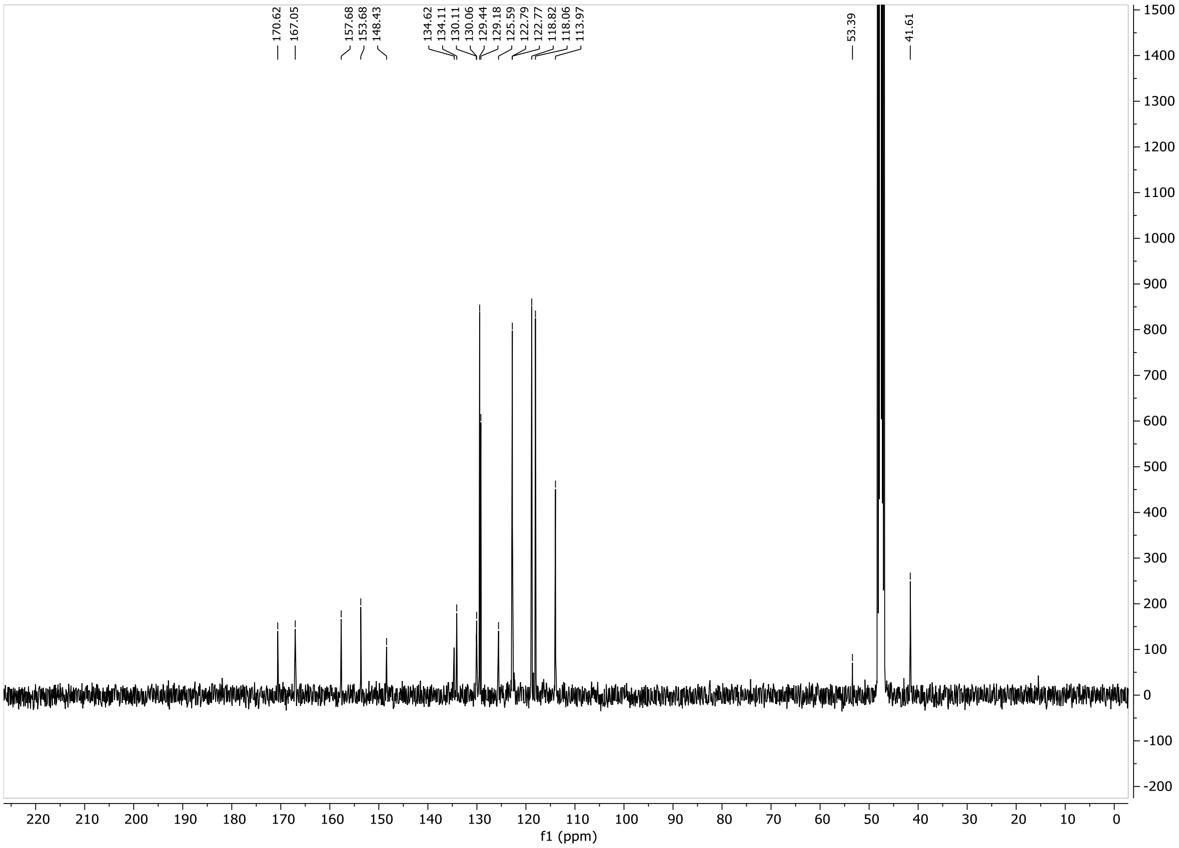

Supporting references

[1] B. Wingelhofer, B. Maurer, E. C. Heyes, A. A. Cumaraswamy, A. Berger-Becvar, E. D. de Araujo, A. Orlova, P. Freund, F. Ruge, J. Park, G. Tin, S. Ahmar, C. H. Lardeau, I. Sadovnik, D. Bajusz, G. M. Keseru, F. Grebien, S. Kubicek, P. Valent, P. T. Gunning, R. Moriggl, *Leukemia* **2018**, *32*, 1135-1146.

[2] Z. Nikolovska-Coleska, R. Wang, X. Fang, H. Pan, Y. Tomita, P. Li, P. P. Roller, K. Krajewski, N. G. Saito, J. A. Stuckey, S. Wang, *Anal. Biochem.* **2004**, *332*, 261-273.

[3] N. Elumalai, A. Berg, K. Natarajan, A. Scharow, T. Berg, *Angew. Chem. Int. Ed.* **2015**, *54*, 4758-4763.

[4] T. Hamada, Y. Okuno, M. Ohmori, T. Nishi, O. Yonemitsu, *Chem. Pharm. Bull.* **1981**, *29*, 128-136.

[5] N. Elumalai, A. Berg, S. Rubner, L. Blechschmidt, C. Song, K. Natarajan, J. Matysik, T. Berg, *Sci. Rep.* **2017**, *7*, 819.

[6] W.-C. Sun, K. R. Gee, D. H. Klaubert, R. P. Haugland, *J. Org. Chem.* **1997**, *62*, 6469-6475.

[7] T. Yamato, T. Arimura, M. Tashiro, *J. Chem. Soc., Perkin Trans. 1* **1987**, 1-7.

[8] S. Caddick, D. B. Judd, A. K. d. K. Lewis, M. T. Reich, M. R. V. Williams, *Tetrahedron* **2003**, *59*, 5417-5423.

[9] M. Gräber, W. Janczyk, B. Sperl, N. Elumalai, C. Kozany, F. Hausch, T. A. Holak, T. Berg, *ACS Chem. Biol.* **2011**, *6*, 1008-1014.

[10] P. A. Ravindranath, S. Forli, D. S. Goodsell, A. J. Olson, M. F. Sanner, *PLoS Comput. Biol.* **2015**, *11*, e1004586.
